# BRCA1 haploinsufficiency for replication stress suppression in primary cells

**DOI:** 10.1038/ncomms6496

**Published:** 2014-11-17

**Authors:** Shailja Pathania, Sangeeta Bade, Morwenna Le Guillou, Karly Burke, Rachel Reed, Christian Bowman-Colin, Ying Su, David T. Ting, Kornelia Polyak, Andrea L. Richardson, Jean Feunteun, Judy E. Garber, David M. Livingston

**Affiliations:** 1 Harvard Medical School, Boston, Massachusetts 02115, USA; 2 Dana-Farber Cancer Institute, Boston, Massachusetts 02215, USA; 3 Stabilité Génétique et Oncogenèse, Université Paris-Sud, CNRS-UMR8200, Gustave-Roussy, Villejuif 94805, France; 4 Department of Hematology/Oncology, Massachusetts General Hospital, Charlestown, Massachusetts 02129, USA; 5 Brigham and Women’s Hospital, Harvard Medical School, 75 Francis St, Boston, Massachusetts 02115, USA

## Abstract

*BRCA1*—a breast and ovarian cancer suppressor gene—promotes genome integrity. To study the functionality of *BRCA1* in the heterozygous state, we established a collection of primary human *BRCA1*^+/+^ and *BRCA1*^mut/+^ mammary epithelial cells and fibroblasts. Here we report that all *BRCA1*^mut/+^ cells exhibited multiple normal *BRCA1* functions, including the support of homologous recombination- type double-strand break repair (HR-DSBR), checkpoint functions, centrosome number control, spindle pole formation, *Slug* expression and satellite RNA suppression. In contrast, the same cells were defective in stalled replication fork repair and/or suppression of fork collapse, that is, replication stress. These defects were rescued by reconstituting *BRCA1*^mut/+^ cells with wt BRCA1. In addition, we observed ‘conditional’ haploinsufficiency for HR-DSBR in *BRCA1*^mut/+^ cells in the face of replication stress. Given the importance of replication stress in epithelial cancer development and of an HR defect in breast cancer pathogenesis, both defects are candidate contributors to tumorigenesis in BRCA1-deficient mammary tissue.

## Abstract (web summary)

BRCA1 is a key breast and ovarian cancer suppressor involved in DSB repair. Here, the authors show that cells heterozygous for several *BRCA1* mutations are universally defective in the response to replication stress, which could contribute to the BRCA1 breast cancer development pathway.

## Graphical abstract

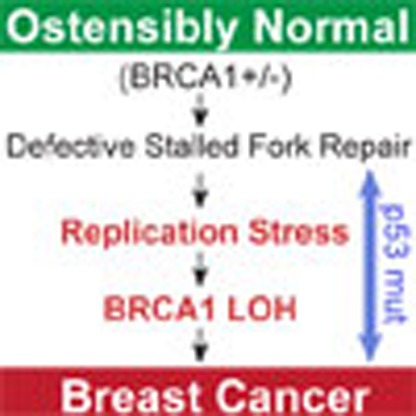

Germ line *BRCA1* mutations increase greatly the risk of breast and ovarian cancer^1^,^2^,^3^. While all cells of males and females with germline *BRCA1* mutations exhibit a heterozygous *BRCA1*^mut/+^ genotype, cancer develops primarily in females, often at young ages and almost exclusively affects the breast and ovaries. Why *BRCA1* is largely a breast and ovarian cancer susceptibility gene, why males are largely protected from *BRCA1* cancer and how an ostensibly normal mammary epithelial cell in a *BRCA1* mutation carrier (*BRCA1*^mut/+^) gives rise to breast cancer cells are largely unknown.

In addition, there is insufficient mechanistic insight into BRCA1 breast tumorigenesis on which to base rational preventive strategies. Their design will, in part, require a deeper appreciation of the biological properties of a heterozygous but ostensibly normal, mammary epithelium (*BRCA1*^mut/+^).

The tumour-suppressing BRCA1 protein is an E3 ubiquitin ligase and a multi-functional scaffold that binds numerous partner proteins^4^,^5^. It plays a key role in genome integrity maintenance, which appears to be an essential component of its tumour-suppressing function^2^,^5^,^6^.

A *BRCA1* loss of heterozygosity (LOH) event is a consistent characteristic of fully developed *BRCA1*-linked tumour cells. Two generic models describe the chain of events that precede it and the concomitant emergence of mammary tumour cells (human mammary epithelial cells (HMECs)). In one, HMECs, despite being heterozygous, are histologically and biologically normal before the emergence of LOH. They fail to exhibit a significant defect in BRCA1 function. Here key events that transform a cell to malignancy follow the loss of all BRCA1 functions at the LOH event and are often preceded by acquisition of a p53 mutation that sustains cell viability in the face of emerging genome disorder^7^.

In the other model, *BRCA1*^mut/+^ HMECs are haploinsufficient for the performance of one or more BRCA1 functions even before any signs of a neoplastic cell phenotype emerge. This model implies that, from the time that mammary epithelial development is complete or at some relatively early time thereafter, *BRCA1*^mut/+^ HMECs cannot perform all BRCA1 genome integrity maintenance functions at normal amplitude. These abnormalities may increase the likelihood that steps in the mammary tumorigenesis process begin long before they become clinically apparent.

In this regard, there is growing evidence of a defect in normal mammary epithelial progenitor differentiation in histologically normal, *BRCA1* heterozygous mammary tissue^8^,^9^,^10^,^11^, implying that the second model is more likely valid than the first. Thus, determining whether *BRCA1* heterozygosity confers haploinsufficiency on HMECs for any of the multiple, known, *BRCA1* functions is a potentially valuable step in achieving a better understanding of *BRCA1* mutation-driven cancer predisposition. In this regard, we have analysed a new collection of primary mammary *BRCA1*^mut/+^ epithelial cells and skin fibroblasts obtained from *BRCA1* mutation carriers for such functions.

## Results

### Primary cell genotyping and lineage determination

Established elements of BRCA1 function were analysed in freshly isolated, morphologically non-neoplastic, primary HMECs and skin fibroblasts derived from multiple *BRCA1*^+/+^ and *BRCA1*^mut/+^ tumour-free women. Twenty-three primary *BRCA1*^mut/+^ fibroblast cultures were derived from skin punch biopsies, and 15 primary *BRCA1*^mut/+^ HMEC cultures were generated from individual prophylactic mastectomy samples (Table 1). HMECs were cultured in serum-free medium.

**Figure 1:**
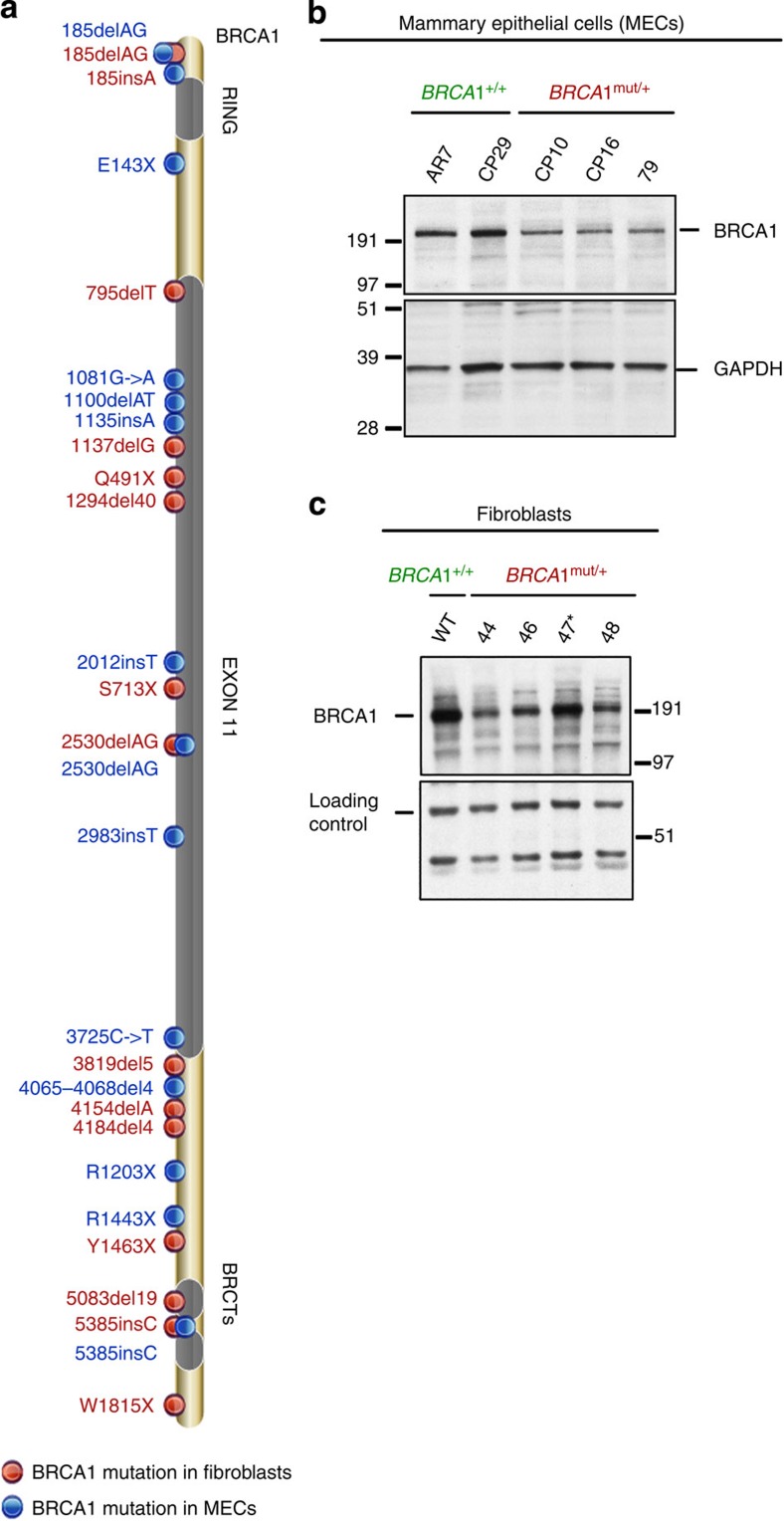
Distribution of *BRCA1* mutations and BRCA1 protein in cells derived from *BRCA1* mutation carriers. (**a**) Cells were derived from skin punch biopsies and prophylactic mastectomies performed on *BRCA1* mutation carrying women. (**b**) Western blot analysis of total BRCA1 protein levels in *BRCA1*^mut/+^ and *BRCA1*^+/+^ HMEC lines. Equivalent amounts of whole-cell lysate (prepared in NETN300) were electrophoresed, blotted and the blots probed with an anti-BRCA1 monoclonal Ab (SD118). GAPDH served as a loading control. (**c**) Western blot analysis of BRCA1 protein levels in the nuclear fraction of *BRCA1*^mut/+^ and *BRCA1*^+/+^ fibroblast strains. Cells were pre-lysed in pre-extraction buffer (PEB, details in Materials and Methods), and the pellet was re-suspended in NETN400 buffer to prepare a nuclear extract. The intense BRCA1 band in 47 (185delAG, marked by an asterisk) is likely the previously discovered truncated product of this mutant allele^45^. A non- specific band served as the loading control.

The properties of *BRCA1*^mut/+^ HMECs were compared with *BRCA1*^+/+^ HMECs (*N*=7), freshly derived from reduction mammoplasty tissue, and those of *BRCA1*^mut/+^ skin fibroblasts with freshly isolated *BRCA1*^*+/+*^ fibroblasts (*N*=11; Table 1). Mutations in *BRCA1* mutant fibroblasts and HMECs were confirmed by homogenous Mass-Extend (hME) analysis^12^ and by direct *BRCA1* gene sequencing (Supplementary Fig. 1a–c). Together, this collection of *BRCA1*^mut/+^ mutations spans nearly the entire *BRCA1* genome (Fig. 1a).

To determine the lineage of cells that grew out of our primary tissue samples under the culturing conditions used, we carried out flow cytometry (FACS)-based analysis of lineage markers (CD44, CD49f, CD24 and EpCAM). In this study, our primary *BRCA1*^mut/+^ and *BRCA1*^+/+^ HMEC cultures were similarly enriched in early basal (CD44^high^, CD24^low^, CD49f^high^ EpCAM^low^) relative to luminal progenitor cells (CD44^low^, CD49f^low^, CD24^high^, EpCAM^high^)^9^,^13^ (Supplementary Fig. 2a). For this analysis, MCF7 was used as a luminal cell line control and MCFDCIS.com as a basal cell line control.

Furthermore, western blot analysis of whole-cell (for HMECs) and nuclear extracts (for fibroblasts) revealed that full-length BRCA1 expression in *BRCA1*^mut/+^ HMEC (Fig. 1b and Supplementary Fig. 2c) and fibroblast strains (Fig. 1c and supplementary Fig. 2b) was lower than that detected in wt *BRCA1*^+/+^ lines. This was in keeping with the proven genetic heterozygosity in these cells. As BRCA1 is much more abundant in S and G2 than in G1, we only analysed wt and heterozygous HMEC and fibroblast cultures that exhibited similar cell cycle profiles and BUdR uptake (see for example, Supplementary Fig. 2c).

### Non-DNA repair-driven BRCA1 genome integrity functions

BRCA1 exhibits two types of genome integrity maintenance functions—those that are directed towards the repair of DNA damage and checkpoint control, and others that sustain genome integrity by contributing to homeostatic functions that are not necessarily driven by DNA damage.

In this context, we asked whether the lower expression of BRCA1 in *BRCA1*^mut/+^ cell cultures was associated with a deficiency in the latter BRCA1 functions. BRCA1 is required for the maintenance of centrosome number^14^, mitotic spindle pole formation^15^,^16^,^17^, mammary development through the regulation of master genes like *Slug*^11^ and heterochromatin-based satellite RNA suppression^18^.

**Figure 2:**
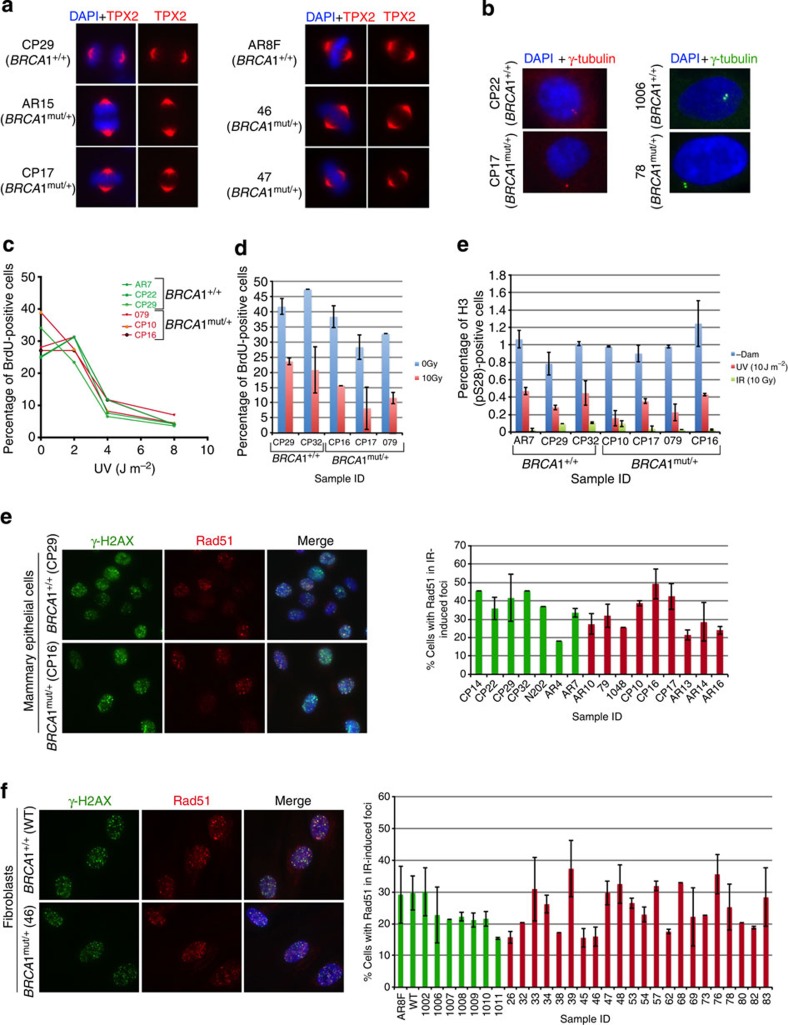
Spindle pole formation, centrosome number, checkpoint activation and Rad51 recruitment to DSB. (**a**) Representative images of HMECs (left panel) and skin fibroblasts (right panel), from *BRCA1*^mut/+^ and *BRCA1*^*+/+*^ were immunostained with an anti- TPX2 Ab to detect spindles; *n*=50 spindles were analysed for each strain. A summary of all strains that were tested in this assay is listed in supplementary Table 1. (**b**) Centrosome number was determined by immunostaining HMECs (left panel) and fibroblasts (right panel) with Ab to γ-tubulin; *n*=50 cells for each line were counted and cells with centrosomes ≤2 were considered normal. A summary of the lines that were tested is presented in Supplementary Table 1. (**c**) S-phase checkpoint in response to UV- and IR-induced DNA damage in control and *BRCA1*^mut/+^ strains. Three *BRCA1*^+/+^ (AR7, CP22 and CP29) and three *BRCA1*^mut/+^ HMEC strains (79, CP10 and CP16) were irradiated with increasing doses of UV (left panel). For IR-induced S-phase checkpoint analysis (right panel), cells were exposed to IR (10 Gy, red). Non-irradiated cells (0 Gy, blue) served as controls. Error bars indicate the s.d. between the results of three, independent experiments. (**d**) G2/M checkpoint activation in response to UV- and IR- induced DNA damage in *BRCA1*^mut/+^ and control cells. *BRCA1*^+/+^ and *BRCA1*^mut/+^ cells were irradiated with either UV (10 J m^−2^) or IR (10 Gy), allowed to recover for 2 h and then harvested for FACS analysis. The percentage of cells in mitosis was determined by staining cells with propidium iodine (PI) and phosphorylated histone H3 (S28) antibody. Mock-irradiated (-Dam) cells served as controls. (**e**) HMECs and (**f**) fibroblasts were exposed to IR (10 Gy) and allowed to recover for 4 h. Cells were fixed and co-immunostained with Abs to γ-H2AX and Rad51. Graphs depicting the fraction of cells with Rad51 foci that co-localized with γ-H2AX foci for each line are plotted for both HMECs and fibroblasts (right panels in **e** and **f**). Mean and s.d. of at least three experiments for each strain are shown. wt *BRCA1*^+/+^ (green) and *BRCA1*^mut/+^ (red) lines.

Each of these functions was compared in heterozygous (*BRCA1*^mut/+^) and control (*BRCA1*^+/+^) cells. Spindle formation was analysed by staining mitotic cells with a TPX2 antibody. No abnormal spindle formation was detected in *BRCA1*^mut/+^ cells (Fig. 2a and Table 1). The effects of robust BRCA1 depletion on this function have been documented^15^.

Similarly, we found that none of the *BRCA1*^mut/+^ and *BRCA1*^+/+^ cells contained greater than 2 centrosomes, implying that centrosome maintenance was normal in these different BRCA1^mut/+^ strains (Fig. 2b and Supplementary Table 1). Although we did not detect any evidence of centrosome amplification in multiple BRCA1 heterozygous cells, other work^7^ with BRCA1 heterozygous tissue has detected a small increase of centrosome amplification (~5%) in the epithelial cells of heterozygous mammary tissue compared with 2.5% in wt tissue.

De-repression of satellite RNA transcription is also a feature of BRCA1 mutant tumours^18^. Furthermore, Brca1 heterozygous cells do not show evidence of satellite de-repression^18^. To test whether this phenotype was present in heterozygous BRCA1 HMECs, two approaches were employed. Quantitative RT-PCR (q-RT-PCR) was performed for alpha satellite variants (SATIII, SATa and mcBox). Satellite RNA transcript levels were also estimated by RNA FISH directed at another satellite RNA, HSATII. Very low levels of satellite RNA were present in primary HMECs, making it difficult to detect any satellite RNA signal by these methods (Supplementary Fig. 3a and b).

To address the effect of *BRCA1* heterozygosity on Slug expression^11^, we compared the Slug level in *BRCA1*^+/+^ and *BRCA1*^mut/+^ HMECs by western blot analysis. In these experiments, MCF7 (a luminal breast cancer line) was used as a negative control and MDA-MB-231 (a basal line) was used as a positive control. No reproducible difference in Slug expression was detected between the *BRCA1*^+/+^ and *BRCA1*^mut/+^ strains that were tested (Supplementary Fig. 3c). Addition of serum^11^ had similar effect on Slug expression in *BRCA1*^+/+^ and *BRCA1*^mut/+^ strains.

### DNA damage checkpoints

BRCA1 plays an important role in regulating both the S phase and G2 checkpoints after DNA damage^19^,^20^. The efficiency of post-damage checkpoint activation was also tested in BRCA1 heterozygous cells. We failed to detect any significant difference in the ability of *BRCA1*^*+/+*^ and ^mut/+^ lines to mount either an S phase (Fig. 2c, left and right panel) or a G2 checkpoint response (Fig. 2d) following IR or UV-induced DNA damage.

### *BRCA1* DNA repair functions double-strand break repair

*BRCA1* plays an essential role in homologous recombination-type double-strand break repair (HR-DSBR)^21^,^22^. Defective HR-DSBR is a well-known property of BRCA1 and related, inherited breast cancers; molecular epidemiology results suggest that it is a risk factor for these cancers^23^,^24^,^25^.

*BRCA1* is attracted to discrete sites of DSB-containing damage, where it directs a complex HR repair response^5^,^26^. Long-standing results show that in *BRCA1*^+/−^ ES cells^27^, HR function is normal until both copies of *BRCA1* are inactivated (*BRCA1*^−/−^). By contrast, others have reported that targeting one copy of *BRCA1* with a mutation (for example, 185delAG) in an established, spontaneously immortal line of human HMECs resulted in a subtle HR defect^28^. Thus, a detailed analysis of multiple, primary human *BRCA1*^mut/+^ and *BRCA1*^+/+^ HMECs and fibroblasts was undertaken to search for evidence of *BRCA1* haploinsufficiency for HR-DSBR in this setting.

Two, well-validated assays were set up to measure HR-DSBR, by testing the recruitment of Rad51 (an indicator of a key step in HR)^29^ to sites of DSBs and by measuring the sensitivity to PARP inhibitors (PI). The first assay clearly showed that *BRCA1*^mut/+^ cells were competent in recruiting Rad51 to sites of DSBs (Figs 2e,f). Moreover, like HR-DSBR-competent cells, they were also insensitive to olaparib (a PI). This assay, described below, relies on the observation that sensitivity to a PI is dependent on the existence of an HR defect^30^. Indeed, *BRCA1* tumour lines (which lack functional *BRCA1* and reveal a defect in HR) are more sensitive to these agents than *BRCA1*^+/+^ cells^31^,^32^.

**Figure 3:**
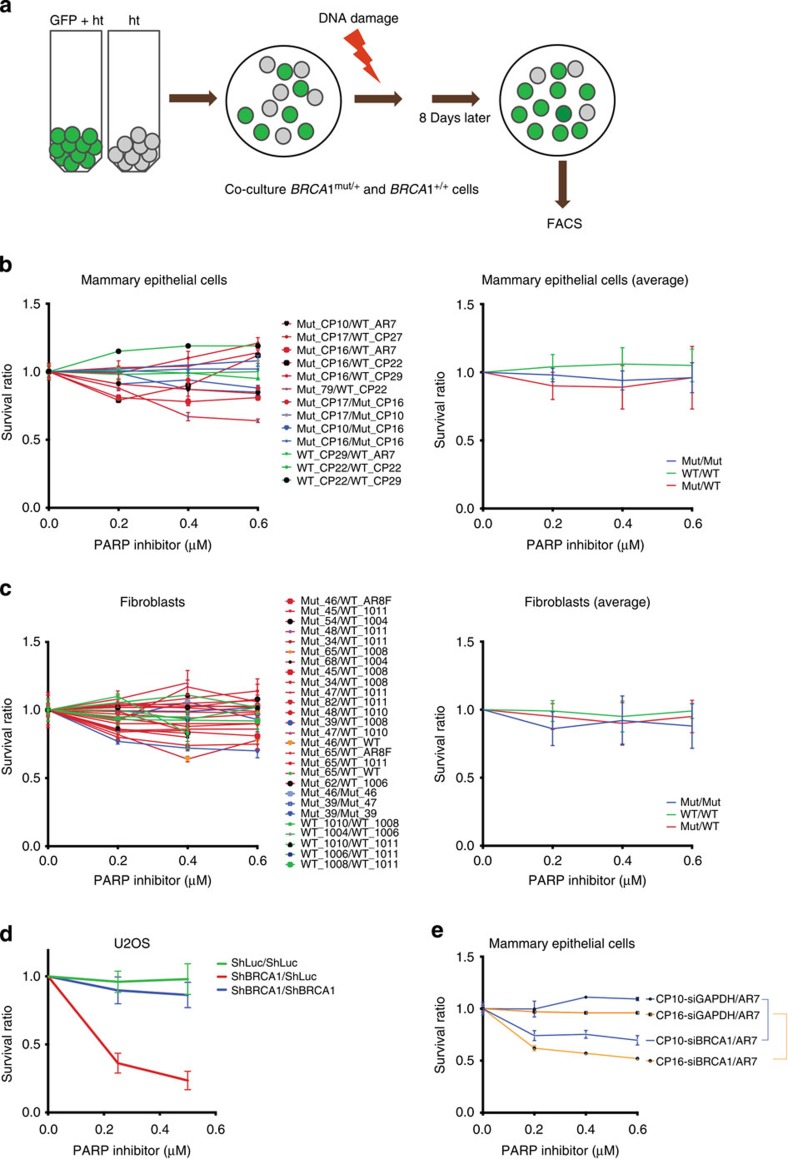
FACS-based cell survival assay shows that HR-DSBR is not defective in *BRCA1*^mut/+^ cells. (**a**) FACS-based cell survival assay was used to determine the sensitivity of cells to various DNA damage inducing agents. *BRCA1*^mut/+^ and *BRCA1*^+/+^ ‘colour-coded’ cells were co-plated and exposed to DNA damaging agents. Cell survival data are plotted as a ratio of GFP positive to GFP negative cells. Ratio between WT/WT(Green), Mutant/Mutant (Blue) and Mutant/WT (Red) is plotted in the graphs below. (**b**) Combinations of *BRCA1*^mut/+^ and *BRCA1*^+/+^ HMECs were exposed to different concentrations of a PARP inhibitor, and the ratio of each of these combinations was plotted (left). The average ratio of WT/WT, Mut/Mut and Mut/WT was also calculated and plotted (right). (**c**) (Left) Combinations of *BRCA1*^mut/+^ and *BRCA1*^+/+^ fibroblasts were exposed to different concentrations of a PARP inhibitor, and the survival ratio of each of these combinations was plotted (left). An average ratio of WT/WT, Mut/Mut and Mut/WT was also calculated and plotted (right). (**d**) U20S cells (containing or lacking a GFP reporter) were infected with ShLuc (control) or ShBRCA1 coding lentiviral vectors. Green=ratio of number of ShLuc-treated cells to ShLuc-treated cells, that is (ShLuc/ShLuc), Blue=ratio of number of ShBRCA1-treated cells to ShBRCA1 treated-cells, that is, ShBRCA1/ShBRCA1 and Red=ratio of number of ShBRCA1-treated cells to ShLuc-treated cells, that is, ShBRCA1/ShLuc. Averages of the results of individual experiments are plotted. (**e**) *BRCA1*^mut/+^ (CP10 and CP16) were transduced with shRNA directed at GAPDH (siGAPDH) or BRCA1 (siBRCA1). Three days post transfection, combinations of siGAPDH or siBRCA1-transduced *BRCA1*^mut/+^ HMECs (CP10 and CP16) were co-plated with AR7 (a *BRCA1*^+/+^ HMEC) and exposed to various doses of a PARP inhibitor. Averages of the results generated by these combinations were plotted. Error bars were calculated as the standard error propagation (SEP) in the ratios of each of the combinations in three independent experiments.

To study the effect of PARP inhibitors in our collection of *BRCA1*^mut/+^ and *BRCA1*^+/+^ cells, a FACS-based cell survival assay of co-cultured cells was employed. Cells were ‘colour-coded’ and tested in pairs, where one cell strain emitted a fluorescent signal (for example, strain A, GFP+) and the other (strain B) did not. Strains A and B were mixed, co-plated and then exposed to a DNA damaging agent of choice. After 7 days of recovery, they were harvested and the relative abundance of each cell population was analysed by FACS (Fig. 3a). The ratio of green/non-green or non-green/green cells reflected the relative survival of the two strains.

When *BRCA1*^mut/+^ and *BRCA1*^+/+^ cells were compared for their sensitivity to olaparib, BRCA1^mut/+^ cells were not found to be demonstrably sensitive (Fig. 3b,c). As a positive control, U20S cell line, made HR-DSBR incompetent by depleting BRCA1 (ShBRCA1 treated), was used along with control (ShLuc treated) cells. Once BRCA1 depleted, these cells proved to be highly sensitive to olaparib, while control cells were not (Fig. 3d). In addition, *BRCA1*^mut/+^ HMEC viability was reduced by olaparib, again only after BRCA1 depletion (si*BRCA1*, Fig. 3e).

Thus, despite the linkage of HR to *BRCA1* breast cancer suppression and in keeping with results obtained in mouse ES cells^27^, these results, too, suggest that *BRCA1*^mut/+^ cells are not defective for HR-dependent DSBR function.

### Stalled replication fork repair

BRCA1 also protects the genome from DNA damage resulting at stalled replication forks^33^,^34^,^35^,^36^. It is rapidly attracted to these damage sites where it joins other proteins that are required for stalled fork repair (SFR). For example, BRCA1 is required for the generation of phospho-RPA32-coated single-stranded DNA (ssDNA), a pre-repair step needed for recruitment of ATRIP/ATR to activate the intra-S and G2/M checkpoints that support SFR^35^,^37^,^38^,^39^.

In the absence of BRCA1, a stalled fork is more likely to be bypassed by translesional synthesis^35^, or, it may collapse into DSB, a hallmark of ‘replication stress’ and an established force in support of epithelial cancer development^40^,^41^. In the mammary epithelium, which undergoes normal periods of extreme proliferation (for example, during pubertal development and/or pregnancy), an accumulation of stalled forks, when not resolved, is likely to result in significant replication stress.

**Figure 4:**
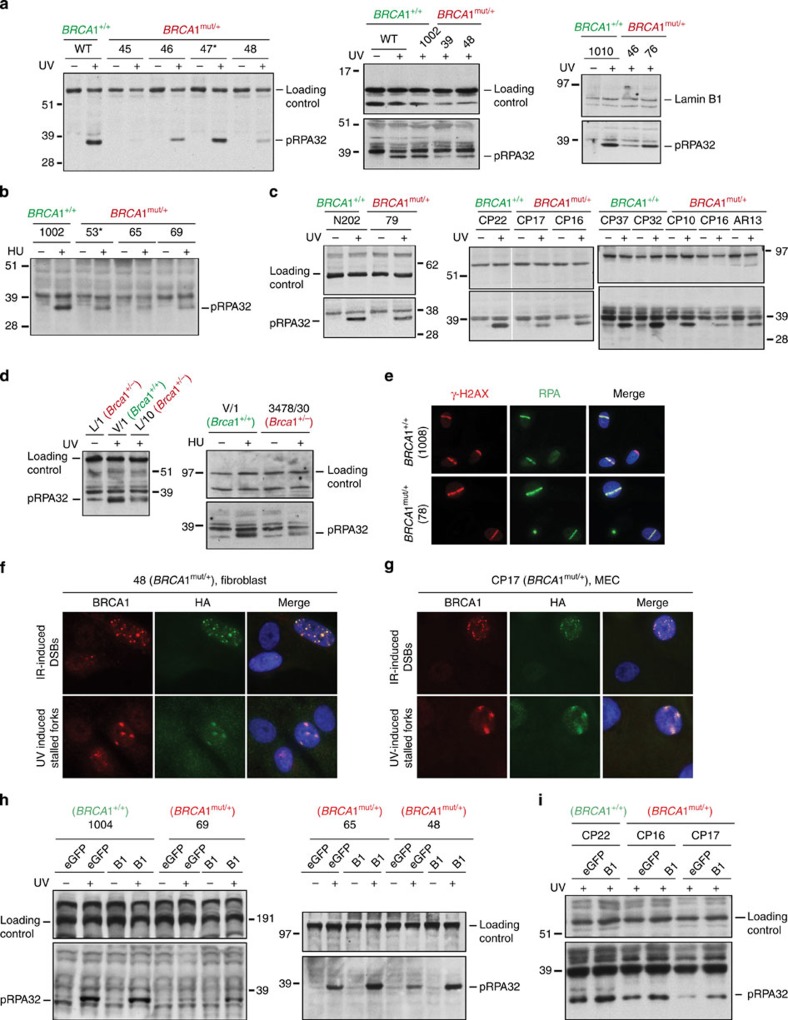
*BRCA1*^mut/+^ cells derived from human and mouse tissue are defective in the generation of phospho-RPA32-coated ssDNA. (**a**) Phospho-RPA32 (pRPA32) loading on chromatin is BRCA1 dependent. After UV-induced DNA damage, *BRCA1*^mut/+^ fibroblasts exhibited reduced pRPA32 loading on ssDNA, compared with *BRCA1*^+/+^ lines. Cells were irradiated with 30 J m^−2^ of UV and harvested 3 h post damage. Chromatin extracts were prepared, and the relevant western blot was probed with an antibody to phosphorylated RPA32 (S4/S8). The replication status for each line was tested on the day of the experiment by BrdU uptake measurement, and only those lines that exhibited similar replication profiles were analysed. A subset of lines tested is shown here. Western blots for other *WT* and *BRCA1* mutant lines are shown in Supplementary Fig. 4b. (**b**) *BRCA1*^mut/+^ fibroblasts reveal reduced pRPA32 loading on ssDNA compared with *BRCA1*^+/+^ lines, after HU exposure (10 mM for 3 h). An asterisk marks strains with the 185delAG mutation. (**c**) *BRCA1*^mut/+^ HMECs reveal reduced pRPA32 loading on ssDNA, compared with *BRCA1*^+/+^ HMECs after UV irradiation. (**d**) Mammary epithelial cells derived from *Brca1*^*+/*−^ (L/10 and 3478/30) and/or *Brca1*^*+/+*^ (V/1) mice were analysed for pRPA32 levels on chromatin after UV- and HU-induced damage. (**e**) *BRCA1*^mut/+^ cells efficiently recruit RPA32 to DSBs. RPA32 loading at laser-induced DSBs was equivalently efficient in *BRCA1*^mut/+^ and *BRCA1*^+/+^ lines. Cells were co-stained with anti- γ-H2AX to reflect the existence of DSBs. (**f**) *BRCA1*^mut/+^ skin fibroblasts (48) and (**g**) mammary epithelial cells (CP17), each infected with a lentiviral vector expressing HA- tagged BRCA1, were either irradiated with 10 Gy IR (upper panel) or 30 J m^−2^ of UV (lower panel). Cells were co-immunostained with Abs to BRCA1 and HA. (**h**,**i**) Phospho-RPA32 recruitment to ssDNA was analysed with a subset of primary *BRCA1*^mut/+^ and *BRCA1*^+/+^ fibroblasts (**h**) and HMECs (**i**), infected with a lentiviral vector expressing either full-length WT BRCA1 (HA-tagged) or eGFP (control). Western blots were immunostained with Ab to phospho-RPA32.

Thus, we asked whether *BRCA1*^mut/+^ cells are haploinsufficient in their ability to support SFR. Employing validated assays, we found that, by comparison with control cells, *BRCA1*^mut/+^ fibroblasts and HMECs were defective in their SFR responses to replication-stalling agents like hydroxyurea (HU) and UV-C (ultraviolet radiation). We have shown previously that, in cells that were heavily depleted of BRCA1, recruitment of phospho-RPA32 (pRPA32) to chromatin was defective in response to UV^35^. This defect was also evident after treatment with HU (Supplementary Fig. 4a). When *BRCA1*^mut/+^ cells were tested for their ability to recruit pRPA32 to chromatin after UV and/or HU treatment, a defect was detected in *BRCA1*^mut/+^ cells (Fig. 4a–c, and Supplementary Fig. 4a–d).

To test whether these abnormal RPA binding observations in *BRCA1*^mut/+^ cells are specifically linked to *BRCA1* haploinsufficiency, we asked whether ectopic wt BRCA1 expression in *BRCA1*^mut/+^ cells corrects them. Infection by a lentiviral BRCA1 coding vector led to wt BRCA1 (HA-tagged) expression in primary *BRCA1*^mut/+^ cells (Fig. 4f,g; Supplementary Fig. 5a). This protein was recruited to DSBs and stalled forks in HMECs and fibroblasts like endogenous wt *BRCA1* (Fig. 4f,g). Its expression suppressed the apparent, post-UV haploinsufficient defect in pRPA32 chromatin recruitment (Fig. 4h,i, respectively). Thus, this defect is a valid representation of *BRCA1* haploinsufficiency.

To test the generality of SFR haploinsufficiency, we isolated MECs from Brca1^+/−^ and Brca1^+/+^ mice. These cells were used to study the generation of phospho-RPA32-coated ssDNA after UV- and HU-induced stalled fork formation. In keeping with results obtained with *BRCA1* heterozygous human cells, we observed reduced phospho-RPA32 coating of ssDNA in *Brca1*^+/−^ mouse cells compared with WT *Brca1*^+/+^ cells (Fig. 4d). This underscores the generality of the finding that cells with one mutated allele for *BRCA1* are haploinsufficient for pRPA32 loading on chromatin.

pRPA32 loading on chromatin is dependent on the generation of ssDNA. Its generation after replication arrest is *BRCA1*-dependent^35^. To detect the generation of ssDNA, a BrdU immunoassay^42^ performed under non-denaturing conditions (HCl) was used. Here, using the same assay, we found that strain 39 (*BRCA1*^mut/+^) generated less ssDNA (Supplementary Fig. 4e,f) compared with strain 1002 (*BRCA1*^+/+^). This supports the hypothesis that *BRCA1*^mut/+^ cells generate less ssDNA, which in turn results in less pRPA32 chromatin loading.

Of note, 1075 (a human *185delAG*/+ strain) failed to exhibit a defect in ssDNA generation. This suggests that the post UV generation of ssDNA was not affected in these cells and explains why we did not observe a defect in pRPA32 loading in them. Possibly, steps downstream of ssDNA generation and pRPA32 loading are defective in *185delAG* strains (see for example, below).

Finally, to test whether the inefficient loading of RPA at stalled forks in *BRCA1*^mut/+^ cells is a reflection of innately reduced RPA activation after DNA damage, we assayed for RPA recruitment to DNA in response to UV laser-induced DSBs. As shown in Fig. 4e, RPA was equivalently recruited to these stripes in *BRCA1*^mut/+^ and +/+ cells. This rules out the possibility of an innate defect in RPA activation after DNA damage.

An inability to form pRPA32-coated ssDNA after DNA damage may result in relevant checkpoint defects^43^. Although we detected an incomplete reduction in pRPA32-coated chromatin after UV-induced DNA damage in *BRCA1*^mut/+^ HMECs, there was no obvious S or G2 checkpoint defect. Thus, incomplete formation of pRPA32-coated ssDNA, in the conditions tested, was, nonetheless, sufficient to initiate a proper checkpoint response.

Given that inefficient loading of pRPA32 on ssDNA is associated with an SFR defect, we asked whether *BRCA1*^mut/+^ strains also experience an abnormally high frequency of collapsed forks compared with their WT counterparts (*BRCA1*^+/+^). Fork collapse can be captured by staining the cells with antibody to 53BP1 and/or γ- H2AX, which is routinely recruited to these damaged structures^35^,^44^.

**Figure 5:**
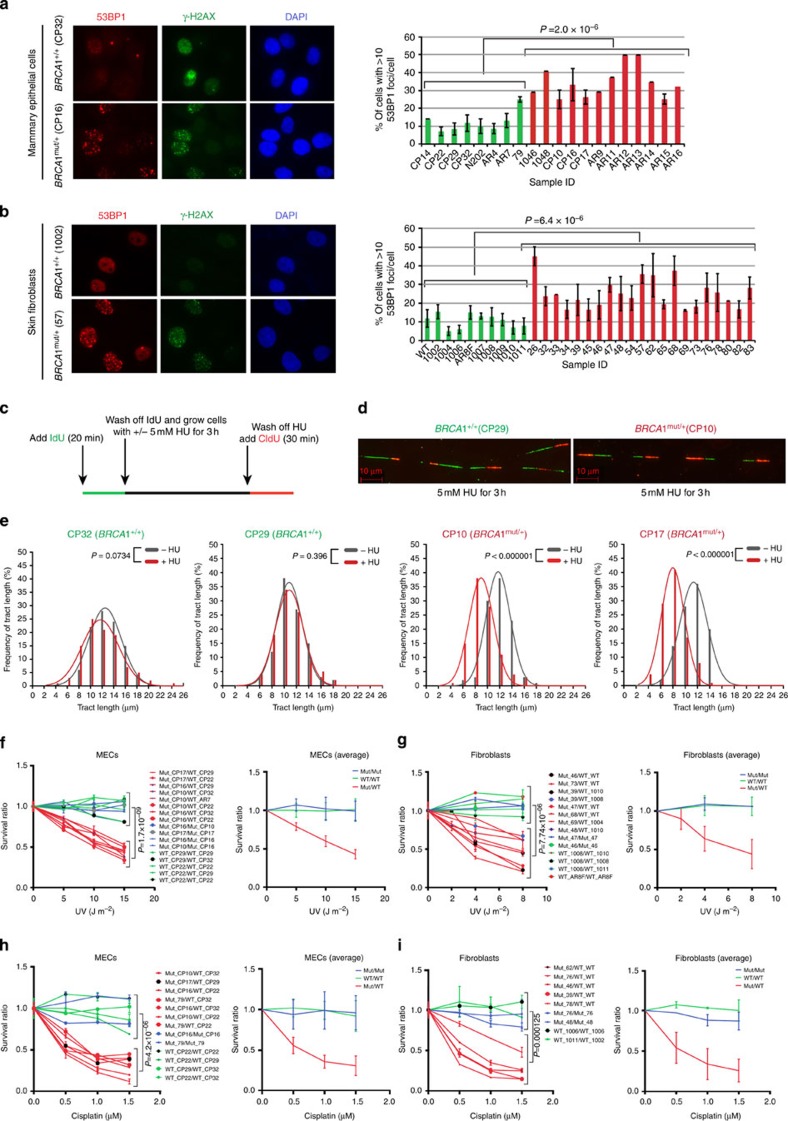
The stalled fork repair pathway is defective in *BRCA1*^*mut/+*^ cells. Heterozygous *BRCA1*^mut/+^ cells reveal increased DNA break formation, after stalled fork-inducing DNA damage, show reduced replication fork stability, and are more sensitive than WT *BRCA1*^+/+^ cells to stalled fork-inducing agents. After exposure to a stalled fork-inducing agent (UV and/or HU), *BRCA1*^mut/+^ cells were prone to increased fork collapse compared with *BRCA1*^+/+^ cells. (**a**) Skin fibroblasts, and (**b**) HMECs derived from *BRCA1* mutation carriers (*BRCA1*^mut/+^) and wild type *BRCA1* counterparts (*BRCA1*^+/+^), were irradiated with low dose UV (5 J m^−2^) and allowed to recover for 18 h. Cells were immunostained with Ab to 53BP1 and γ- H2AX (a marker for collapsed replication forks). The right (R) panel depicts the percentage of cells with ≥10 53BP1 foci per cell in HMECs and fibroblasts. Mean and s.d. of at least three experiments for each strain are shown (green: wt *BRCA1*^+/+^; red: *BRCA1*^mut/+^). (**c**) Schematic representation of DNA fibre experiment. (**d**) Representative tracts from DNA fibre experiments with HMECs (*BRCA1*^*+/+*^ , CP29; *BRCA1*^*mut/+*^, CP10) treated with 5 mM HU for 3 h. Green and red tracts correspond to IdU and CldU incorporation, respectively. Red scale bar represents 10 μm length. (**e**) Distribution curves of IdU tract lengths in the presence and absence of HU (5 mM for 3 h) for both *BRCA1*^*+/+*^ (first two plots, CP32 and CP29) and *BRCA1*^*mut/+*^ (last two plots, CP10 and CP17) cells. Red and Grey curves represent the presence and absence of HU in the culture medium, respectively. At least 200 tracts were scored for each distribution curve. (**f**,**g**) (Left panels) Combinations of *BRCA1*^mut/+^ and *BRCA1*^+/+^ HMECs (**f**) and fibroblasts (**g**) were irradiated with different doses of UV. (Right) Average of data plotted on left. (**h**) Combinations of *BRCA1*^mut/+^ and *BRCA1*^+/+^ HMECs and (**i**) fibroblasts were incubated with increasing concentrations of cisplatin for 15 h. Cells were allowed to recover for 6 days and then harvested for FACS analysis. Panels on the right show the averages of data plotted on the left.

*BRCA1*^mut/+^ cells, stained 18 h post UV with p-S1778 53BP1 and γ-H2AX Abs, revealed an increase in fork collapse by comparison with wt controls (Fig. 5a,b). This again implies that the efficiency of SFR is compromised in *BRCA1*^mut/+^ cells, leading to higher fork collapse and incomplete resolution/repair of these structures. Thus, *BRCA1* is haploinsufficient for the suppression of replication stress in primary HMECs and fibroblasts.

Of note, *185delAG*-bearing strains (that is, 26, 47, 53, 57, AR19L) exhibited near normal loading of pRPA32 onto chromatin (marked with asterisk in Fig. 4a and Supplementary Fig. 4b), but more abundant 53BP1 foci by comparison with control cells (Fig. 5b). Others have shown that the *185delAG* allele expresses a modestly truncated BRCA1 protein, translation of which is initiated immediately downstream of the mutation near the 5′ end of the gene^45^. Thus, one might hypothesize that *185delAG* is a hypomorph, capable of supporting some but not all BRCA1 SFR support functions.

To better understand the fate of collapsed forks in *BRCA1*^mut/+^ cells, we carried out DNA fibre analysis. We wished to assess two phenotypes: (1) the stability of nascent replication tracts after fork stalling and (2) the efficiency of replication restart. Cells were pulse labelled with IdU for 20 min followed by treatment with or without 5 mM HU for 3 h. After washing off HU, cells were incubated in the presence of CldU for 30 min (Fig. 5c). This protocol allows the analysis of the fate of nascent replication tracts (synthesized before HU addition) during replication stalling, as well as replication fork restart after a replication block is eliminated (Fig. 5c).

DNA fibre assays have been used previously with *Brca1*^−/−^ mouse ES cells to show that BRCA1 is required to suppress degradation of nascent strands after replication stalling induced by HU treatment^36^. In that study, replication restart was not affected by the absence of BRCA1.

In keeping with these results, we find that, in the presence of HU, *BRCA1*^mut/+^ cells exhibited increased degradation of the nascent strand (shorter green tracts) at stalled forks compared with the *BRCA1*^+/+^ cells (Fig. 5d,e; Supplementary Fig. 5c). As shown in Fig. 5e, the distribution of nascent DNA tract lengths (green tracts, Fig. 5d) for *BRCA1*^+/+^ MECs (CP32 and CP29) was not different between HU-treated and -untreated samples (red and grey curves, respectively). However, the red curves shifted towards shorter lengths (increased degradation) after treatment with HU in *BRCA1*^mut/+^ cells (CP10 and CP17). By contrast, no significant difference in the ability of *BRCA1*^mut/+^ cells to restart replication was detected after replication stress had abated (Fig. 5c–e; Supplementary Fig. 5c).

These results further support our conclusion that the stability of stalled forks is compromised in *BRCA1* heterozygous (*BRCA1*^mut/+^) cells.

To assess further the conclusion that inefficient SFR in *BRCA1*^mut/+^ cells results in increased DNA breaks, we employed comet assays. In UV-treated cells there was a greater increase in DNA breaks in *BRCA1*^mut/+^ when compared with *BRCA1*^+/+^ cells (Supplementary Fig. 5d,e). This result reaffirms the finding that, faced with replication stalling, *BRCA1* heterozygous primary cells exhibited signs of replication stress, unlike *BRCA1*^+/+^ cells.

### Roles of *BRCA1*-associated proteins in SFR in *BRCA1* ^mut/+^ cells

A stalled fork serves as a scaffold to recruit and concentrate proteins that play critical role/s in stabilizing, processing, repairing and restarting a stalled fork. This is essential to prevent the risk of its collapse into a DSB, a prime contributor to genomic instability. We tested a subset of the proteins that are known to be recruited to a stalled fork, with an eye towards those that interact and/or function together with BRCA1 to carry out SFR.

Specifically, we asked whether recruitment of Rad51, a BRCA1 partner in HR-based DSBR^46^,^47^ and a protein known to play an HR-independent repair role at stalled forks^48^, is affected in *BRCA1*^mut/+^ cells. We found that Rad51 recruitment to UV-induced stalled forks was reduced in *BRCA1*^mut/+^ compared with *BRCA1*^+/+^ cells (Supplementary Fig. 6b). This was not surprising, given that Rad51 is recruited to RPA-coated ssDNA, the generation of which is compromised in *BRCA1*^mut/+^ cells. We also found that the same *BRCA1*^mut/+^ strains that revealed efficient Rad51 recruitment to DSBs (Fig. 2e,f) were defective in recruiting Rad51 to stalled forks. This implies that the role of Rad51 at a stalled fork is different from that at a DSB and further confirms the observations made by other groups who found that Rad51 helps restart stalled forks in an HR-independent manner^36^,^48^,^49^. In addition, Scully *et al.*^50^ detected significant differences between the mechanism of repair at a non replication fork-associated DSB and at a stalled fork-induced break.

We next assayed the efficiency of CtIP recruitment to UV-induced stalled forks. CtIP is an established BRCA1 partner^51^ and plays an important role in replication restart after stalled fork formation^52^,^53^. We found previously that BRCA1 is required for the recruitment of CtIP to UV-induced stalled forks^35^. In light of this evidence, we asked whether CtIP recruitment is compromised in *BRCA1*^mut/+^ cells. Just as for Rad51, *BRCA1*^mut/+^ cells exhibited reduced CtIP recruitment to sites of UV-induced fork stalling (Supplementary Fig. 6a). It is unclear whether this defect in CtIP recruitment to stalled forks is a direct result of a reduced BRCA1 protein level or reduced pRPA32-coated ssDNA. Nonetheless, these data further confirm that *BRCA1*^mut/+^ cells are defective in SFR.

Finally, we addressed the fate of Mre11 at stalled forks in *BRCA1*^mut/+^ and *BRCA1*^+/+^ cells. Mre11 is a BRCA1-associated nuclease that has been implicated in helping restart collapsed and stalled replication forks via resection and initiation of repair at these sites^49^,^54^,^55^. Given that *BRCA1*^mut/+^ cells exhibit reduced ssDNA generation, defective pRPA32 loading on chromatin and collapsed forks, we asked whether Mre11 recruitment mirrors this phenotype. The data revealed increased Mre11 recruitment to the sites of UV-induced stalled forks in *BRCA1*^mut/+^ cells compared with *BRCA1*^+/+^ cells (Supplementary Fig. 6c). Given that Mre11 is a nuclease that is recruited to DSBs, it seems reasonable to propose that increased fork collapse in *BRCA1*^mut/+^ cells results in DSBs, that, in turn, recruit Mre11. Thus, *in lieu* of possibilities discussed above, increased Mre11 recruitment to UV-induced stalled forks in *BRCA1*^mut/+^ cells may be yet another indicator of the reduced stability of stalled forks in *BRCA1*^mut/+^ cells.

### Cell sensitivity to different DNA damage inducing agents

In an effort to validate the observation that primary *BRCA1*^mut/+^ cells are defective for SFR and suppression of replication stress, the relative sensitivity of these primary cells to stalled fork-inducing agents, like UV and cisplatin^56^, was tested in differentially coloured, co-cultured cells. In multiple comparisons of primary *BRCA1*^mut/+^ and *BRCA1*^+/+^ fibroblasts and HMECs, the heterozygotes were significantly more sensitive than the wt cells to UV (Fig. 5e,f) and cisplatin (Fig. 5g,h).

### HR-DSBR in *BRCA1* ^mut/+^ cells undergoing replication stress

The discordance between multiple intact and one defective BRCA1-associated functions in numerous, primary heterozygous cell strains suggests that *BRCA1*^mut/+^ cells can preferentially direct their limited stores of intact BRCA1 protein to checkpoint activation, HR-DSBR, centrosome, SLUG control and spindle pole function, and less effectively to SFR. Alternatively, less BRCA1 protein is required for the former functions than the latter one. In either case, we asked whether, when these cells encounter sufficient replication stress, BRCA1 becomes preferentially dedicated to SFR and, in doing so, the pool of BRCA1 available for otherwise intact functions is reduced. If it falls sufficiently, do *BRCA1*^mut/+^ cells now become multiply haploinsufficient, that is, for other known *BRCA1* functions that were formerly intact in these cells.

**Figure 6:**
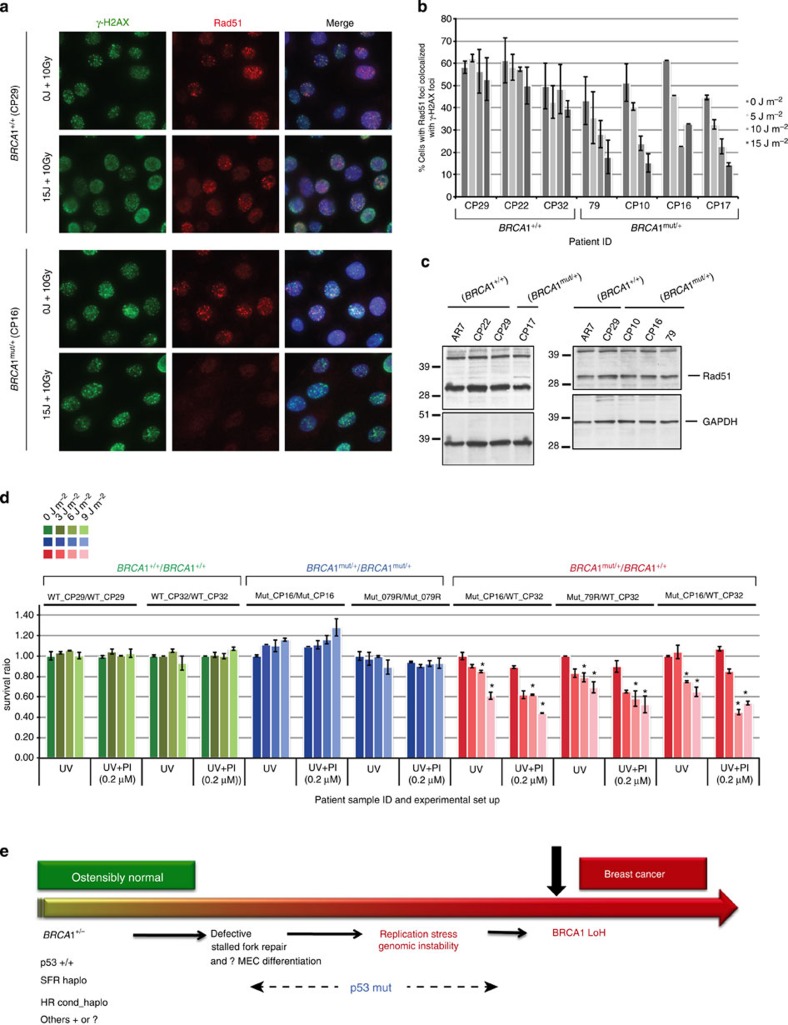
Evidence of conditional haploinsufficiency for DSBR in *BRCA1*^mut/+^ HMECs after pre-exposure to a stalled fork- inducing agent. (**a**) Recruitment of Rad51 to IR-induced DSBs is reduced in heterozygous *BRCA1*^mut/+^, and not in WT *BRCA1*^+/+^ HMECs, when pre-exposed to stalled fork-inducing damage. HMECs derived from a *BRCA1* mutation carrier (CP16, *BRCA1*^mut/+^) and a wt counterpart (CP29, *BRCA1*^+/+^) were irradiated with different doses of UV (5, 10 or 15 J m^−2^) and allowed to recover for 1 h. Cells were then irradiated with IR (10 Gy) and fixed 4 h post IR. Fixed cells were coimmunostained with Abs to γ-H2AX and Rad51. Additional wt and heterozygous strains were also assayed (in panel **b**). (**b**) Additional *BRCA1*^+/+^ and *BRCA1*^mut/+^ strains were analysed as described in (**a**). A graph depicting the fraction of cells in each additional HMEC strain that contains Rad51 foci after exposure to increasing doses of UV followed by 10 Gy dose of IR was plotted. The mean results and s.d. of data from at least three experiments are shown for each line. (**c**) Rad51 expression in *BRCA1*^mut/+^ and *BRCA1*^+/+^ HMEC lines. Whole-cell extracts from various *BRCA1*^mut/+^ and *BRCA1*^+/+^ strains were analysed by western blot. GAPDH was used as a loading control in these blots. (**d**) Combinations of *BRCA1*^mut/+^ and *BRCA1*^+/+^ HMECs (green: *BRCA1*^+/+^/*BRCA1*^+/+^, blue: *BRCA1*^mut/+^/ *BRCA1*^mut/+^ and red: *BRCA1*^mut/+^/*BRCA1*^+/+^) were irradiated with different doses of UV (0, 3, 6 and 9 J m^−2^), allowed to recover for 1 h, and then treated with either 0.2 μM PARP inhibitor (PI=olaparib; UV+PI) or DMSO as control (UV). Cells were grown for five more days before harvesting for FACS analysis. Data are plotted for the three, different cell combinations, and the error bars were calculated as the standard error propagation (SEP) in the ratios of each of the combinations in three, independent experiments. Data marked with an asterisk (*) reveal statistically significant differences (*P*-value<0.05) between UV and UV+PI sets. (**e**) One Possible Model of *BRCA1* mutation- driven tumorigenesis. This model speculates that certain abnormal developments might occur during the extended period between full mammary development and the appearance of a *BRCA1* breast cancer.

To address this possibility, we pre-exposed cells to increasing doses of UV and then assayed them for other *BRCA1* functions (other than SFR). To assay for HR, the UV-treated cells were irradiated with IR and analysed for recruitment of Rad51 to DSBs (Fig. 6a). To assay for spindle formation and centrosome maintenance, we allowed the cells to recover for one and/or two full cycles of cell division and then analysed the cells for spindles as well as centrosomes.

As shown in Fig. 2e,f, multiple *BRCA1*^mut/+^ and *BRCA1*^+/+^ cell strains recruited Rad51 to IR-induced DSBs with equal efficiency in the absence of UV pre-treatment. However, the ability of *BRCA1*^mut/+^ cells to recruit Rad51 to DSBs became increasingly defective after exposure to increasing doses of UV (Fig. 6a,b). No such effect was detected in *BRCA1*^+/+^ cells. We asked whether changes in BRCA1 protein levels in the UV pre-treated heterozygotes could account for reduced Rad51 recruitment, but no obvious alterations were observed (Supplementary Fig. 4e). This result, along with the observation that Rad51 protein levels in *BRCA1*^mut/+^ and *BRCA1*^+/+^ were also similar (Fig. 6c), suggests that a defect in Rad51 recruitment to DSBs, in UV- pretreated *BRCA1*^mut/+^ cells, is a result of a defect in the ability of a limited pool of BRCA1 protein to respond to DSBs by driving the HR-DSBR process.

To assess further the apparent emergence of ‘conditional haploinsufficiency’ for HR-DSBR in the presence of replication stress, we used the FACS-based assay described earlier to determine the survival efficiency of *BRCA1*^mut/+^ cells in the presence of olaparib. The question here was whether pre-exposure of cells to stalled fork-inducing damage (for example, UV) compromises the ability of these cells to carry out DSBR. If so, the *BRCA1*^mut/+^ cells should become olaparib-sensitive. Evidence presented in Fig. 6d showed this to be the case. Exposure of *BRCA1*^mut/+^ cells to increasing doses of UV before adding olaparib rendered them acutely sensitive to a relatively low concentration of olaparib (Fig. 6d).

Centrosome number and spindle formation in the same cell strains were not altered under these conditions (data not shown). This implies that, at the very least, there is conditional haploinsufficiency^57^ for HR-DSBR in *BRCA1*^mut/+^ cells facing sufficient replication stress.

## Discussion

Multiple primary fibroblast and HMECs derived from non-tumour tissue of *BRCA1* mutation carriers reveal, for the first time, the existence of *BRCA1* haploinsufficiency for one of its established, genome integrity maintenance functions, that is, its ability to support SFR and to prevent replication stress. By contrast, no such defect was detected among several other such functions. Conceivably, haploinsufficiency for some of these apparently unaffected BRCA1 functions occurs but takes considerably longer to develop during the life of a *BRCA1*^mut/+^ individual than did the defect in SFR. In addition, the quantity of BRCA1 needed to sustain at least some of its other functions may be significantly less than that required for this activity.

Furthermore, in keeping with a model first proposed by Bartek *et al.*^57^, the data presented here also reveal a possible hierarchy of DNA repair functions in *BRCA1*^mut/+^ cells, wherein a defect in SFR, if not resolved, can trigger an otherwise undetectable defect in HR-DSBR following enhanced replication stalling. In effect, representative primary *BRCA1*^mut/+^ HMECs and fibroblasts exhibited a state of innate haploinsufficiency for SFR and ‘conditional’ haploinsufficiency for HR-DSBR.

Thus, in keeping with the model of Bartek *et al.*^57^, we hypothesize that, when the amplitude of replication stalling rises above a threshold level in cells that are already deprived of a full complement of intact BRCA1, the available BRCA1 pool is dedicated first to preventing and repairing collapsed forks. This leaves even less BRCA1 available to form complexes that are required for the execution of HR at DSB that are not associated with fork collapse. The latter effect can be hypothesized to give rise to the *de novo* development of an HR defect. This prediction was borne out experimentally.

Why replication stress did not affect any other *BRCA1* genome integrity maintenance function, other than HR-DSBR, is unknown. One possible explanation is that BRCA1 appears to execute each of these unaffected functions as a member of a large, multi-subunit complex(es)^4^,^15^,^21^,^58^,^59^. Conceivably, these complexes are sufficiently stable, well-compartmentalized and function efficiently enough before the onset of replication stalling that they are not disadvantaged by such an event.

Given the strong contributory history of inadequate SFR to epithelial cancer development and the fact that it is, thus far, the only apparent haploinsufficient *BRCA1* DNA repair abnormality, we speculate that SFR haploinsufficiency serves as an early and persistent contributor to the long process that gives rise to *BRCA1* breast cancer (Fig. 6e). The fact that, when exposed to sufficient levels of replication stress, conditional *BRCA1* HR-DSBR haploinsufficiency emerged in *BRCA1* heterozygous cells suggests that this defect might join SFR haploinsufficiency as a *BRCA1* breast cancer co-contributor in mammary epithelial progenitor cells that are experiencing sufficient ongoing replication stress. This would befit its widely accepted role as a major *BRCA1* breast cancer risk factor.

Assuming that other *BRCA1* functions remain to be discovered, it is conceivable that one or more of them, too, is haploinsufficient in the cells we have analysed. Thus, the current picture, while new, may be incomplete.

*BRCA1* haploinsufficiency for SFR was also apparent in *Brca1*+/− mouse MECs. Given that *Brca1*+/− mice are not tumour prone^60^, this suggests that haploinsufficiency for SFR in mMECs is not sufficient to drive tumorigenesis, especially given the short life span of mice. Furthermore, despite the tissue specificity (breast and ovary) of *BRCA1* mutant cancer, a haploinsufficient phenotype was not limited to mammary epithelial cells. Similar defects were also displayed by *BRCA1*^mut/+^ fibroblasts, thereby supporting the notion that multiple factors combine to generate the tissue specificity of *BRCA1*-mutant cancer.

Indeed, while drafting this manuscript, a new haploinsufficient role for *BRCA1* was reported^61^. The authors showed that transcription of the *CYP1A* gene, which encodes an estrogen-metabolizing enzyme, is upregulated in *BRCA1* heterozygous cells^61^. In addition, Savage *et al.*^61^ showed that these oestrogen metabolites result in increased DNA damage in *BRCA1* heterozygous cells. In light of the existence of defective SFR in *BRCA1* heterozygous cells, it is reasonable to predict that such a defect would be a key avenue through which haploinsufficiency for oestrogen metabolite detoxification could result in DNA damage.

Even if SFR haploinsufficiency was not the only DNA repair defect in *BRCA1* heterozygous HMECs, the high potential for it to give rise to chronic replication stress may well be clinically significant. This is because chronic replication stress is an established and common force in human epithelial cancer formation^40^,^41^,^62^,^63^.

Moreover, others have observed defects in differentiation in populations of primary *BRCA1*^mut/+^ HMECs^8^,^9^,^10^,^11^. As yet undefined *BRCA1* functional abnormalities underlie this set of phenotypes and could, when deciphered, enlarge the results described here. The extent to which SFR and, possibly, conditional HR-DSBR haploinsufficiency contribute to them is unknown but worthy of investigation. DNA damage is known to perturb the differentiation of certain cell types^64^.

Recently, Winqvist *et al.*^65^ reported the existence of haploinsufficiency for replication stress responsiveness in EBV-immortalized B lymphocytes and primary T cells derived from PALB2 heterozygotes. Their ability to perform HR was not analysed. These data obtained from cells with a single PALB2 mutant genotype represent an example of haploinsufficiency for a known BRCA1- and BRCA2- interacting protein that is also a breast cancer suppressor. Thus, those results and evidence reported here imply that haploinsufficiency in replication stress suppression is a feature of ostensibly normal mammary epithelial cells of two, different sets of mutation carriers.

In this regard, evidence of *BRCA1* haploinsufficiency was sought by Buchholz *et al.*^66^ in *BRCA1* heterozygous fibroblasts and lymphocytes, and by Konishi *et al.*^28^ in a human HMEC line where a *BRCA1* mutation (185delAG) was introduced into one allele by gene targeting. Buchholz *et al.*^66^ observed increased sensitivity of *BRCA1* heterozygous fibroblasts to ionizing radiation (IR) and increased chromatid breaks in lymphocytes after IR. Given the pleiotropic effect of IR on DNA (for example, strand breaks, fork stalling, base damage, DNA-adducts^67^,^68^,^69^), one cannot rule out that the sensitivity to IR is a result of contribution of multiple forms of DNA damage and not just a response to DSB formation.

Similarly, in Konishi *et al.*^28^ it was suggested that the targeted heterozygous clones were defective in HR-DSBR. Although increased sensitivity of these clones to IR and a reduced HR-DSBR signal in HR reporter-containing cells were detected, they, like we, failed to observe any sensitivity of their *BRCA1*^mut/+^ cells to PARP inhibition, raising a question regarding the existence of an HR defect. Given that the test cells were reported to be slow to proliferate, this could have contributed towards apparent HR deficiency.

The possibility that persistent replication stress is a tumour-promoting force in *BRCA1*^mut/+^ mammary epithelial cells offers, a hypothetical, mechanism-based route to *BRCA1* breast cancer prevention. If specific subsets of *BRCA1*^mut/+^ HMECs normally advance beyond the manifestation of an SFR defect to develop additional *BRCA1* functional deficiencies accompanied by a much higher risk of tumorigenicity, their selective elimination might suppress subsequent *BRCA1* breast cancer development.

## Methods

### Isolation and culture of human MECs and fibroblasts from tissue biopsies

Tissue samples were briefly washed in PBS and then minced and digested overnight at 37 °C in medium containing 1 mg ml^−1^ of collagenase type III (Roche). For digestion, MEGM medium (Lonza) was used for breast tissue, and Dulbecco’s modified Eagle’s Medium (DMEM) with 5% fetal bovine serum (FBS) for skin tissue. The digested tissue was pelleted and fibroblasts were cultured in DMEM supplemented with 15% FBS (Gibco), 1% Pen/Strep (Gibco) and 1% Glutamine (Gibco), and HMECs were grown in MEGM medium supplemented with 1% Pen/Strep.

### Isolation and culture of mouse MECs from mouse mammary tissue

Primary mouse MEC cultures were generated from the 4th and 5th pairs of mammary fat pads using a sterile technique. The tissue was digested overnight at 37 °C in serum-free Leibovitz-15 medium containing 3 mg ml^−1^ of collagenase A (Sigma). Digestion was stopped by adding 1 × volume of 10% serum containing DMEM. The pelleted cells and organoids were plated and cultivated in DMEM/F12 50:50 medium supplemented with 10% FCS, 50 units ml^−1^ penicillin, 50 μg ml^−1^ streptomycin (Life Technologies), 5 μg ml^−1^ recombinant human insulin (Sigma), 5 ng ml^−1^ recombinant human EGF (Sigma) and 5 ng ml^−1^ cholera toxin (Calbiochem).

### Mouse model and genotyping

The mouse Brca1 null allele used in this study was generated by crossing mice harbouring the BRCA1F5-13 conditional allele (kindly provided by Dr Jos Jonker’s group)^60^ with Meox2-Cre deleter mice purchased from Jackson Labs (Bar Harbor, ME—Stock #003755). Mouse genotyping was performed on genomic DNA extracted from mouse-tail snips using standard procedures^60^. Genotyping for Cre-generated Brca1 null allele was carried out with primers GenoB1-A (5′-AGGTACCAGTTATGAGTTAGTCGTGTGCCTGAGTCA-3′) and GenoB1-D (5′-GGCTACCTATAACTACTCTCTAACAACGAAGTGCAA-3′), which yielded a 654-bp fragment. The wt brca1 allele was genotyped using primers GenoB1-A and GenoB1-B (5′-GCTGAGATTAAAGTGCAGGCCACCACACTCAGTGAT-3′), which yielded a PCR product of 495 bp for the wt allele and 624 bp for the floxed allele. PCR amplification conditions used were as described previously^60^. Primers Meox2Cre1 (5′-CCTGAAAGCAGTTCTCTGGGACCACCTTCTTTTGGCTTC-3′) and Meox2Cre2 (5′-CTTCTTCTTGGGTCCTCCCAGATCCTCCTCAGAAATCAGC-3′) were used to verify the presence of Meox2 Cre allele. Amplified fragment was 423 bp.

### Transfection, infection and selection

For siRNA experiments, cells were grown in six-well plates and transfected with 100 pmoles of siRNA with RNAiMAX (Invitrogen) according to the manufacturer’s protocol. Where relevant, experiments were initiated 48 h after transfection. All siRNA oligonucleotides were purchased from Thermo Scientific. siRNA oligonucleotides used were siBRCA1 (On Target Plus BRCA1, catalog number CTM-41735) and siGAPDH (On Target Plus GAPDH, catalog number D-001830-01-20). For shRNA experiments, shRNA encoding lentiviruses were generated using 293FT-packaging cells in the presence of lipofectamine (Invitrogen). Cells infected with puroR-encoding lentiviruses were selected transiently using 2.5 μg ml^−1^ puromycin (Santa Cruz). ShBRCA1 and shLuc were acquired from The RNAi Consortium (TRC). The target sequence for shBRCA1 was 5′-AGAATCCTAGAGATACTGAA-3′. For BRCA1 reconstitution experiments, lentiviral packaging plasmids, VSVG and PSPAX, were used to package BRCA1 and/or eGFP plasmids in 293FT cells using lipofectamine (Invitrogen). Cells were infected with the lentivirus and selected using 6 μg ml^−1^ of Blasticidin (Invitrogen). For colour-coding experiments, hTERT and GFPhTERT containing retroviruses were prepared by packaging the plasmids pMIG-hTERT and pBABE-hygro-hTERT with the retrovirus packaging plasmids, pMD-MLV and pMD-G, in 293FT cells. hTERT-infected cells were selected with hygromycin B (Roche) (50 μg ml^−1^).

### Immnoblotting and antibodies

Whole-cell extracts were prepared by lysing cells in NETN300 lysis buffer (300 mM NaCl, 20 mM Tris–HCl buffer pH7.8, 0.5% NP-40, 1 mM EDTA) for 1 h at 4 °C. Nuclear extracts were prepared by pre-extracting the cytoplasmic protein fraction by incubating the cells in pre-extraction buffer, that is, PEB (0.5% Triton -X-100, 20 mM HEPES pH 7.0, 100 mM NaCl, 3 mM MgCl_2_ and 300 mM Sucrose). Incubation was carried out at 4 °C for 20 min. Cells were pelleted, washed once in PEB, and lysed in NETN 400 lysis buffer (400 mM NaCl, 20 mM Tris-HCl buffer pH7.8, 0.5% NP-40, 1 mM EDTA) for 45 min at 4 °C. All lysis buffers were supplemented with 1 × protease inhibitor (Roche) and Halt Phosphatase inhibitor (Thermo Scientific). Chromatin extracts were prepared as described previously^43^. Immunoprecipitation for HA- tagged BRCA1 was carried out by incubating whole-cell extracts with an HA antibody (Covance) for 2 h, followed by 1 h incubation with Protein A beads (GE healthcare) at 4°C. The beads were washed in NETN 150 buffer (150 mM NaCl, 20 mM Tris–HCl buffer pH7.8, 0.5% NP-40, 1 mM EDTA). Antibodies used for western blotting were phospho-RPA32 (Bethyl Labs, A300-245A; 1:2,000), BRCA1 (SD118; 1:1,000), GAPDH (Santa Cruz, SC-25778; 1:4,000), pS53BP1-S25 (Novus Biologicals, NB100-1803; 1:5,000), Rad51 (Santa Cruz, SC-8349; 1:600), Slug (Cell Signaling, C19G7; 1:3,000), Vinculin (Santa Cruz, SC-55465; 1:1,000), BRCA1 (MS110; 1:1,000) and HA (Covance, MMS-101P; 1:4,000). Uncropped western blots for Figs 1,4,6, and Supplementary Figs 2–5 are shown in Supplementary Fig. 7.

### Immunofluorescence and antibodies

Cells on coverslips were fixed with 4% paraformaldehyde/2% Sucrose for 15 min, and triton extracted (0.5% Triton X-100 in PBS) for 4 min. Cells were blocked with 5%BSA/PBST and then incubated with respective antibodies for 30 min at 37 °C followed by incubation with secondary antibodies (FITC or Rhodamine) for 30 min at 37 °C. Primary antibodies used in immunofluorescence studies were BRCA1 (Upstate; 1:500), phospho-53BP1(S1778) (Cell Signaling, 2675S; 1:200), RPA (Cal Biochem, NA13; 1:100), 53BP1 (Bethyl Labs, A300-272A; 1:2,000), Rad51(Santa Cruz, SC-8349; 1:150), Mre11 (Genetex, GTX70212 1:200), CtIP (generous gift from Dr. Richard Baer), HA (Covance, MMS-101P; 1:500) and γ-H2AX (Millipore, 05-636; 1:5,000). For TPX2 (Bethyl Labs, A300-429A; 1:400) and γ-tubulin (Sigma- Aldrich, T6557; 1:1,000) staining, the cells were pre-fixed with acetone:methanol (3:7) at −20 °C for 10 min, followed by triton extraction (0.2% triton-X-100 in 20 mM HEPES, pH 7.4, 50 mM NaCl, 3 mM MgCl2, 300 mM Sucrose) at room temperature. Primary and secondary antibody staining was carried out as described above.

### Cell treatments

For analysis of phospho-RPA32 loading on chromatin, cells were treated with stalled fork inducing agents like HU (Sigma) and/or UV. Cells were incubated in HU (10 mM)-containing medium for 4 h before harvesting for further analysis. For UV treatment, cells were irradiated with 30 J m^−2^ UV with a 254 nm UV-C lamp (UVP Inc., Upland, CA) and harvested 4 h post UV. UV-irradiation through a micropore membrane was performed as described previously^43^. For colour- coded FACS-based cell survival assays, the Parp inhibitor, olaparib (Selleck), was added at final concentrations of 0.2, 0.4 and 0.6 μM for 6 days. cisplatin (Novaplus) was added at final concentrations of 0.5, 1.0 and 1.5 μM for 24 h. Medium was replaced, and the cells were allowed to grow for five more days. The doses of UV used were 5, 10 and 15 J m^−2^, and cells were allowed to recover for 6 days before they were harvested for FACS analysis. Laser-induced DNA breaks were generated as described in Greenberg *et al.*^4^

### DNA fibre assay

DNA fibres were prepared and analysed as described previously^48^,^49^ with a few modifications. In brief, cells were labelled with 25 μM IdU for 20 min, washed two times and incubated in presence of 5 mM HU for 3 h. This was followed by incubation with 250 μM CldU for 30 min. Labelled cells were harvested, mixed 1:5 with unlabelled cells, lysed and spread on slides to obtain single-DNA tracts. After fixation, denaturation and blocking, the DNA tracts were stained with rat anti-CldU (Abcam, ab6326), followed by staining with a secondary antibody Alexa fluor 555 goat anti-rat overnight at 4 °C. DNA tracts were then stained with mouse anti- IdU (BD Biosciences, 555627) followed by a secondary antibody Alexa- 488 goat anti mouse. ImageJ software was used for determining the tract lengths based on scale bar generated during microscopy.

### Detection of ssDNA (BrdU ssDNA Assay)

BrdU ssDNA assay was performed as described previously^42^. In brief, cells on coverslips were cultured with 30 mM BrdU for 20 h, and then released in BrdU-free medium for 16 h. Cells were stained as described previously^42^.

### Sequencing and hME

Cells lines were sequenced to confirm their mutations via direct sequencing or by the hME sequencing method. Genomic DNA was prepared from Blood and a DNeasy kit (Qiagen), and a mutation locus-specific PCR reaction was carried out to amplify the region of interest. For direct sequencing, the amplified PCR products were purified using a Qiagen PCR purification kit and were sent for sequencing. For hME analysis, a locus-specific primer extension reaction of the PCR amplified region was carried out in the presence of a mixture of di-deoxy and deoxy NTPs. Allele-specific extension products were analysed by mass spectrometry to determine the specific sequence. More details of the protocol are available at the following link: http://cancer-seqbase.uchicago.edu/documents/AssayDesign3.1Guide.pdf

### Comet assay and analysis

For detection of DNA breaks, alkaline comet assays were performed using the Single-Cell Gel Electrophoresis Assay kit (Trevigen) according to the manufacturer’s instructions. The quantification of tail DNA was carried out using CellProfiler software.

### Flow cytometry, checkpoints and colour-coding-based cell survival FACS assay

For cell cycle analysis, cells were pulse-labelled with 10 μM BrdU for 30 min (for HMECs) and 1.5 h (for fibroblasts) in respective culture media. Single-cell suspensions were fixed in 70% ice-cold ethanol. Cells were incubated with an anti-BrdU FITC conjugate antibody (Becton Dickinson, 1:10 dilution made in Blocking solution from Thermo Scientific) at room temperature in the dark for 45 min. Finally, the cells were resuspended in propidium iodide and RNAse staining buffer (Becton and Dickinson) and analysed using a Becton Dickinson FACS (Mountain View, CA).

For checkpoint assays, cells were irradiated with UV and/or IR and allowed to recover for 2 h. For S-phase checkpoint analysis, cells were incubated with BrdU, as described above, before harvesting and fixing for FACS analysis. For G2 checkpoint, fixed cells were incubated with an Alexa Fluor anti-phospho-histone H3 (Ser10) antibody diluted in 2% BSA/PBS at room temperature in the dark for 2 h. Cells were washed and resuspended in propidium iodide and RNAse-containing staining buffer.

For colour-coded FACS-based assays, GFP-positive and -negative cells were mixed in equal numbers (8,000 cells per strain) and plated in 6 cm^2^ plates. After drug and/or UV treatment, cells were allowed to recover for 6 days before being harvested for FACS analysis.

### Satellite RNA q-RT-PCR

Cells grown in 6 cm^2^ plates were collected, RNA was prepared using an RNeasy Plus Mini Kit (Qiagen), followed by cDNA preparation. q-RT-PCR was carried out with primers for SatA, SatIII, mcbox and β-Actin. More details and primer sequences are described in Zhu *et al.*^18^

## Author contributions

D.M.L., S.P., J.E.G. and J.F. conceptualized the study. S.P. and D.M.L. designed the experiments and wrote the manuscript. S.P. performed the experiments, analysed the data and oversaw the experiments carried out by S.B., R.R. and K.B. Tissue from *BRCA1* mutation carriers and non-mutation carriers was collected under the guidance of J.E.G. A.L.R. helped provide breast tissue, and K.P. provided some of the MEC strains for the MEC collection. M.G. helped derive some of the MEC strains and carried out western blot analysis for SLUG. M.G. and Y.S. assisted in determining the lineage of the MEC strains by FACS analysis. C.B.-C. provided the mice to carry out breedings for *Brca1*^+/−^ mouse MEC-based experiments and also assisted in statistical analysis of the cell sensitivity assays. D.T.T. helped with satellite RNA FISH experiments. All authors read and contributed to editing the manuscript.

## Additional information

**How to cite this article**: Pathania, S. *et al.* BRCA1 haploinsufficiency for replication stress suppression in primary cells. *Nat. Commun.* 5:5496 doi: 10.1038/ncomms6496 (2014).

## Supplementary Material

Supplementary Information Supplementary Figures 1-7, Supplementary Table 1 and Supplementary References.

## Figures and Tables

**Table 1:** Primary fibroblast and HMEC strains (*BRCA1* ^+/+^ and *BRCA1* ^mut/+^) used in this study.

**Number**	**Study ID**	**Procedure**	**Age**	**Mutation**
*Fibroblasts*				
1	26	Skin Punch Biopsy	38	185delAG
2	32	Skin Punch Biopsy	55	185delAG
3	33	Skin Punch Biopsy	56	185delAG
4	34	Skin Punch Biopsy	29	Y1463X
5	39	Skin Punch Biopsy	50	S713X
6	45	Skin Punch Biopsy	49	5083del19
7	46	Skin Punch Biopsy	26	1137delG
8	47	Skin Punch Biopsy	48	185delAG
9	48	Skin Punch Biopsy	32	4184del4
10	53	Skin Punch Biopsy	52	185delAG
11	54	Skin Punch Biopsy	53	4154delA
12	57	Skin Punch Biopsy	27	185delAG
13	62	Skin Punch Biopsy	38	1294del40
14	65	Skin Punch Biopsy	36	3819del5
15	68	Skin Punch Biopsy	46	Q491X
16	69	Skin Punch Biopsy	30	5385insC
17	73	Skin Punch Biopsy	37	795delT
18	76	Skin Punch Biopsy	43	2530delAG
19	78	Skin Punch Biopsy	42	W1815X
20	80	Skin Punch Biopsy	49	185insA
21	82	Skin Punch Biopsy	62	185delAG
22	83	Skin Punch Biopsy	45	IVS19+1G>A
23	1075	Skin Punch Biopsy	26	185delAG
				
1	WT	Skin Punch Biopsy	56	N/A
2	1002	Skin Punch Biopsy	33	N/A
3	1004	Skin Punch Biopsy	58	N/A
4	1006	Skin Punch Biopsy	43	N/A
5	1007	Skin Punch Biopsy	50	N/A
6	1008	Skin Punch Biopsy	48	N/A
7	1009	Skin Punch Biopsy	69	N/A
8	1010	Skin Punch Biopsy	46	N/A
9	1011	Skin Punch Biopsy	58	N/A
10	AR8F	Reduction Mammo	45	N/A
11	AR20L	Reduction Mammo	31	N/A
				
*Mammary Epithelial Cells*				
1	79	Proph. Mx	41	E143X
2	1046	Proph. Mx	28	3725C>T
3	1048	Proph. Mx	50	185delAG
4	CP10	Proph. Mx	43	1135insA
5	CP16	Proph. Mx	45	4065-4068del
6	CP17	Proph. Mx	28	2012insT
7	AR1	Proph. Mx	46	R1443X
8	AR9	Proph. Mx	34	1100delAT
9	AR10	Proph. Mx	28	1081G->A
10	AR11	Proph. Mx	37	5385insC
11	AR12	Proph. Mx	48	R1203X
12	AR13	Proph. Mx	26	5385insC
13	AR14	Proph. Mx	38	5385insC
14	AR15	Proph. Mx	44	2530delAG
15	AR16	Proph. Mx	41	2983insT
				
1	CP14	Reduc. Mammo.	31	N/A
2	CP22	Reduc. Mammo.	49	N/A
3	CP29	Reduc. Mammo.	28	N/A
4	CP32	Reduc. Mammo.	38	N/A
5	AR4	Reduc. Mammo.	25	N/A
6	AR7	Reduc. Mammo.	33	N/A
7	N202	Reduc. Mammo.	27	N/A

N/A, not applicable.

Twenty-three primary fibroblast strains were derived from skin punch biopsies and 15 primary, mammary epithelial cell (HMECs) strains from prophylactic mastectomies (Proph. Mx) performed on *BRCA1* mutation carrying (*BRCA1*^mut/+^) women. One primary fibroblast strain (1075) was derived from breast skin tissue obtained during prophylactic mastectomy. *BRCA1*^+/+^ control HMECs (*n*=7) were derived from reduction mammoplasty tissue (Reduc. Mammo.), and control fibroblasts (*n*=11) were derived from skin punch biopsies and reduction mammoplasties from women lacking *BRCA1* mutations.

## References

[b1] King M.-C., Marks J. H., Mandell J. B. & Group N.Y.B.C.S. Breast and ovarian cancer risks due to inherited mutations in BRCA1 and BRCA2. Science 302, 643–646 (2003). 14576434 10.1126/science.1088759

[b2] Narod S. A. & Foulkes W. D. BRCA1 and BRCA2: 1994 and beyond. Nat. Rev. Cancer. 4, 665–676 (2004). 15343273 10.1038/nrc1431

[b3] Walsh T. & King M.-C. Ten genes for inherited breast cancer. Cancer Cell 11, 103–105 (2007). 17292821 10.1016/j.ccr.2007.01.010

[b4] Greenberg R. A. *et al.* Multifactorial contributions to an acute DNA damage response by BRCA1/BARD1-containing complexes. Genes Dev. 20, 34–46 (2006). 16391231 10.1101/gad.1381306 PMC1356099

[b5] Huen M. S. Y., Sy S. M. H. & Chen J. BRCA1 and its toolbox for the maintenance of genome integrity. Nat. Rev. Mol. Cell Biol. 11, 138–148 (2009). 20029420 10.1038/nrm2831 PMC3899800

[b6] Silver D. P. & Livingston D. M. Mechanisms of BRCA1 Tumor Suppression. Cancer Discov. 2, 679–684 (2012). 22843421 10.1158/2159-8290.CD-12-0221 PMC3437262

[b7] Martins F. C. *et al.* Evolutionary pathways in BRCA1-associated breast tumors. Cancer Discov. 2, 503–511 (2012). 22628410 10.1158/2159-8290.CD-11-0325 PMC3738298

[b8] Burga L. N. *et al.* Altered proliferation and differentiation properties of primary mammary epithelial cells from BRCA1 mutation carriers. Cancer Res. 69, 1273–1278 (2009). 19190334 10.1158/0008-5472.CAN-08-2954 PMC3041511

[b9] Lim E. *et al.* Aberrant luminal progenitors as the candidate target population for basal tumor development in BRCA1 mutation carriers. Nat. Med. 15, 907–913 (2009). 19648928 10.1038/nm.2000

[b10] Liu S. *et al.* BRCA1 regulates human mammary stem/progenitor cell fate. Proc. Natl Acad. Sci. USA 105, 1680–1685 (2008). 18230721 10.1073/pnas.0711613105 PMC2234204

[b11] Proia T. A. *et al.* Genetic predisposition directs breast cancer phenotype by dictating progenitor cell fate. Cell Stem Cell 8, 149–163 (2011). 21295272 10.1016/j.stem.2010.12.007 PMC3050563

[b12] Thomas R. K. *et al.* High-throughput oncogene mutation profiling in human cancer. Nat. Genet. 39, 347–351 (2007). 17293865 10.1038/ng1975

[b13] Keller P. J. *et al.* Mapping the cellular and molecular heterogeneity of normal and malignant breast tissues and cultured cell lines. Breast Cancer Res. 12, R87 (2010). 20964822 10.1186/bcr2755 PMC3096980

[b14] Kais Z. *et al.* KIAA0101 interacts with BRCA1 and regulates centrosome number. Mol. Cancer Res. 9, 1091–1099 (2011). 21673012 10.1158/1541-7786.MCR-10-0503 PMC3157549

[b15] Joukov V. *et al.* The BRCA1/BARD1 heterodimer modulates ran-dependent mitotic spindle assembly. Cell 127, 539–552 (2006). 17081976 10.1016/j.cell.2006.08.053

[b16] Parvin J. D. The BRCA1-dependent ubiquitin ligase, gamma-tubulin, and centrosomes. Environ. Mol. Mutagen. 50, 649–653 (2009). 19274767 10.1002/em.20475

[b17] Pujana M. A. *et al.* Network modeling links breast cancer susceptibility and centrosome dysfunction. Nat. Genet. 39, 1338–1349 (2007). 17922014 10.1038/ng.2007.2

[b18] Zhu Q. *et al.* BRCA1 tumour suppression occurs via heterochromatin-mediated silencing. Nature 477, 179–184 (2011). 21901007 10.1038/nature10371 PMC3240576

[b19] Xu B., St K. & Kastan M. B. Involvement of Brca1 in S-phase and G(2)-phase checkpoints after ionizing irradiation. Mol. Cell Biol. 21, 3445–3450 (2001). 11313470 10.1128/MCB.21.10.3445-3450.2001 PMC100266

[b20] Kastan M. B. & Bartek J. Cell-cycle checkpoints and cancer. Nature 432, 316–323 (2004). 15549093 10.1038/nature03097

[b21] Zhong Q. *et al.* Association of BRCA1 with the hRad50-hMre11-p95 complex and the DNA damage response. Science 285, 747–750 (1999). 10426999 10.1126/science.285.5428.747

[b22] Moynahan M. E., Chiu J. W., Koller B. H. & Jasin M. Brca1 controls homology-directed DNA repair. Mol. Cell 4, 511–518 (1999). 10549283 10.1016/s1097-2765(00)80202-6

[b23] Walsh T. & King M.-C. Ten genes for inherited breast cancer. Cancer Cell 11, 103–105 (2007). 17292821 10.1016/j.ccr.2007.01.010

[b24] Venkitaraman A. R. Cancer susceptibility and the functions of BRCA1 and BRCA2. Cell 108, 171–182 (2002). 11832208 10.1016/s0092-8674(02)00615-3

[b25] Scully R. & Livingston D. M. In search of the tumour-suppressor functions of BRCA1 and BRCA2. Nature 408, 429–432 (2000). 11100717 10.1038/35044000 PMC2981135

[b26] Zhang J. & Powell S. N. The role of the BRCA1 tumor suppressor in DNA double-strand break repair. Mol. Cancer Res. 3, 531–539 (2005). 16254187 10.1158/1541-7786.MCR-05-0192

[b27] Moynahan M. E., Cui T. Y. & Jasin M. Homology-directed dna repair, mitomycin-c resistance, and chromosome stability is restored with correction of a Brca1 mutation. Cancer Res. 61, 4842–4850 (2001). 11406561

[b28] Konishi H. *et al.* Mutation of a single allele of the cancer susceptibility gene BRCA1 leads to genomic instability in human breast epithelial cells. Proc. Natl Acad. Sci. USA 108, 17773–17778 (2011). 21987798 10.1073/pnas.1110969108 PMC3203756

[b29] West S. C. Molecular views of recombination proteins and their control. Nat. Rev. Mol. Cell Biol. 4, 435–445 (2003). 12778123 10.1038/nrm1127

[b30] Bryant H. E. *et al.* Specific killing of BRCA2-deficient tumours with inhibitors of poly(ADP-ribose) polymerase. Nature 434, 913–917 (2005). 15829966 10.1038/nature03443

[b31] Rottenberg S. *et al.* High sensitivity of BRCA1-deficient mammary tumors to the PARP inhibitor AZD2281 alone and in combination with platinum drugs. Proc. Natl Acad. Sci. USA 105, 17079–17084 (2008). 18971340 10.1073/pnas.0806092105 PMC2579381

[b32] Helleday T., Bryant H. E. & Schultz N. Poly(ADP-ribose) polymerase (PARP-1) in homologous recombination and as a target for cancer therapy. Cell Cycle 4, 1176–1178 (2005). 16123586 10.4161/cc.4.9.2031

[b33] Fang J., Chen T., Chadwick B. P., Li E. & Zhang Y. Ring1b-mediated H2A ubiquitination associates with inactive X chromosomes and is involved in initiation of X inactivation. J. Biol. Chem. 279, 52812–52815 (2004). 15509584 10.1074/jbc.C400493200

[b34] Li L. BRCA1 forks over new roles in DNA-damage response- before and beyond the breaks. Mol. Cell 44, 174–176 (2011). 22017867 10.1016/j.molcel.2011.10.003 PMC3324970

[b35] Pathania S. *et al.* BRCA1 is required for postreplication repair after UV-induced dna damage. Mol. Cell 44, 235–251 (2011). 21963239 10.1016/j.molcel.2011.09.002 PMC3200447

[b36] Schlacher K., Wu H. & Jasin M. A distinct replication fork protection pathway connects fanconi anemia tumor suppressors to RAD51-BRCA1/2. Cancer Cell 22, 106–116 (2012). 22789542 10.1016/j.ccr.2012.05.015 PMC3954744

[b37] Zou L. & Elledge S. J. Sensing DNA damage through ATRIP recognition of RPA-ssDNA complexes. Science 300, 1542–1548 (2003). 12791985 10.1126/science.1083430

[b38] Toledo L. I. *et al.* ATR prohibits replication catastrophe by preventing global exhaustion of RPA. Cell 155, 1088–1103 (2013). 24267891 10.1016/j.cell.2013.10.043

[b39] Cimprich K. A. & Cortez D. ATR: an essential regulator of genome integrity. Nat. Rev. Mol. Cell Biol. 9, 616–627 (2008). 18594563 10.1038/nrm2450 PMC2663384

[b40] Bartkova J. *et al.* DNA damage response as a candidate anti-cancer barrier in early human tumorigenesis. Nature 434, 864–870 (2005). 15829956 10.1038/nature03482

[b41] Gorgoulis V. G. *et al.* Activation of the DNA damage checkpoint and genomic instability in human precancerous lesions. Nature 434, 907–913 (2005). 15829965 10.1038/nature03485

[b42] Rubbi C. P. & Milner J. Analysis of nucleotide excision repair by detection of single-stranded DNA transients. Carcinogenesis 22, 1789–1796 (2001). 11698340 10.1093/carcin/22.11.1789

[b43] Cortez D. Unwind and slow down: checkpoint activation by helicase and polymerase uncoupling. Genes Dev. 19, 1007–1012 (2005). 15879550 10.1101/gad.1316905 PMC1360198

[b44] Harding S. M. & Bristow R. G. Discordance between phosphorylation and recruitment of 53BP1 in response to DNA double-strand breaks. Cell Cycle 11, 1432–1444 (2012). 22421153 10.4161/cc.19824

[b45] Buisson M., Anczuków O., Zetoune A. B., Ware M. D. & Mazoyer S. The 185delAG mutation (c.68_69delAG) in the BRCA1 gene triggers translation reinitiation at a downstream AUG codon. Hum. Mutat. 27, 1024–1029 (2006). 16941470 10.1002/humu.20384

[b46] Cousineau I., Abaji C. & Belmaaza A. BRCA1 regulates RAD51 function in response to DNA damage and suppresses spontaneous sister chromatid replication slippage: implications for sister chromatid cohesion, genome stability, and carcinogenesis. Cancer Res. 65, 11384–11391 (2005). 16357146 10.1158/0008-5472.CAN-05-2156

[b47] Scully R. *et al.* Association of BRCA1 with Rad51 in mitotic and meiotic cells. Cell 88, 265–275 (1997). 9008167 10.1016/s0092-8674(00)81847-4

[b48] Petermann E., Orta M. L., Issaeva N., Schultz N. & Helleday T. Hydroxyurea-stalled replication forks become progressively inactivated and require two different RAD51-mediated pathways for restart and repair. Mol Cell. 37, 492–502 (2010). 20188668 10.1016/j.molcel.2010.01.021 PMC2958316

[b49] Schlacher K. *et al.* Double-strand break repair-independent role for BRCA2 in blocking stalled replication fork degradation by MRE11. Cell 145, 529–542 (2011). 21565612 10.1016/j.cell.2011.03.041 PMC3261725

[b50] Willis N. A. *et al.* BRCA1 controls homologous recombination at Tus/Ter- stalled mammalian replication forks. Nature 510, 556–559 (2014). 24776801 10.1038/nature13295 PMC4118467

[b51] Wong A. K. *et al.* Characterization of a carboxy-terminal BRCA1 interacting protein. Oncogene 17, 2279–2285 (1998). 9811458 10.1038/sj.onc.1202150

[b52] Duquette M. L. *et al.* PLOS genetics: CtIP is required to initiate replication-dependent interstrand crosslink repair. PLoS Genet. 8, e1003050 (2012). 23144634 10.1371/journal.pgen.1003050 PMC3493458

[b53] Yeo J. E., Lee E. H., Hendrickson E. & Sobeck A. CtIP mediates replication fork recovery in a FANCD2-regulated manner. Hum. Mol. Genet. 23, 3695–3705 (2014). 24556218 10.1093/hmg/ddu078 PMC4065146

[b54] Trenz K., Smith E. J., Smith S. & Costanzo V. ATM and ATR promote Mre11 dependent restart of collapsed replication forks and prevent accumulation of DNA breaks. EMBO J. 25, 1764–1774 (2006). 16601701 10.1038/sj.emboj.7601045 PMC1440833

[b55] Hashimoto Y., Puddu F. & Costanzo V. RAD51- and MRE11-dependent reassembly of uncoupled CMG helicase complex at collapsed replication forks. Nature Structural &amp. Mol. Biol. 19, 17–24 (2012). 10.1038/nsmb.2177 PMC4306020 22139015

[b56] Dronkert M. L. & Kanaar R. Repair of DNA interstrand cross-links. Mutat. Res. 486, 217–247 (2001). 11516927 10.1016/s0921-8777(01)00092-1

[b57] Bartek J., Lukas J. & Bartkova J. DNA damage response as an anti-cancer barrier: damage threshold and the concept of ‘conditional haploinsufficiency’. Cell Cycle 6, 2344–2347 (2007). 17700066 10.4161/cc.6.19.4754

[b58] Bochar D. A. *et al.* BRCA1 is associated with a human SWI/SNF-related complex: linking chromatin remodeling to breast cancer. Cell 102, 257–265 (2000). 10943845 10.1016/s0092-8674(00)00030-1

[b59] Wang B. *et al.* Abraxas and RAP80 form a BRCA1 protein complex required for the DNA damage response. Science 316, 1194–1198 (2007). 17525340 10.1126/science.1139476 PMC3573690

[b60] Liu X. *et al.* Somatic loss of BRCA1 and p53 in mice induces mammary tumors with features of human BRCA1-mutated basal-like breast cancer. Proc. Natl Acad. Sci. USA 104, 12111–12116 (2007). 17626182 10.1073/pnas.0702969104 PMC1924557

[b61] Savage K. I. *et al.* BRCA1 deficiency exacerbates estrogen induced DNA damage and genomic instability. Cancer Res. 74, 2773–2784 (2014). 24638981 10.1158/0008-5472.CAN-13-2611 PMC4024319

[b62] Halazonetis T. D., Gorgoulis V. G. & Bartek J. An oncogene-induced DNA damage model for cancer development. Science 319, 1352–1355 (2008). 18323444 10.1126/science.1140735

[b63] Negrini S., Gorgoulis V. G. & Halazonetis T. D. Genomic instability—an evolving hallmark of cancer. Nat. Rev. Mol. Cell Biol. 11, 220–228 (2010). 20177397 10.1038/nrm2858

[b64] Sherman M. H., Bassing C. H. & Teitell M. A. Regulation of cell differentiation by the DNA damage response. Trends. Cell Biol. 21, 312–319 (2011). 21354798 10.1016/j.tcb.2011.01.004 PMC3089693

[b65] Nikkilä J. *et al.* Heterozygous mutations in PALB2 cause DNA replication and damage response defects. Nat. Commun. 4, 2578 (2013). 24153426 10.1038/ncomms3578 PMC3826652

[b66] Buchholz T. A. *et al.* Evidence of haplotype insufficiency in human cells containing a germline mutation in BRCA1 or BRCA2. Int. J. Cancer 97, 557–561 (2002). 11807777 10.1002/ijc.10109

[b67] Dextraze M.-E., Gantchev T., Girouard S. & Hunting D. DNA interstrand cross-links induced by ionizing radiation: an unsung lesion. Mutat. Res. 704, 101–107 (2010). 20079875 10.1016/j.mrrev.2009.12.007

[b68] Hagen U. Current aspects on the radiation induced base damage in DNA. Radiat. Environ. Biophys. 25, 261–271 (1986). 3547457 10.1007/BF01214639

[b69] Nikjoo H., O’Neill P., Wilson W. E. & Goodhead D. T. Computational approach for determining the spectrum of DNA damage induced by ionizing radiation. Radiat. Res. 156, 577–583 (2001). 11604075 10.1667/0033-7587(2001)156[0577:cafdts]2.0.co;2

