# Supplementary material for: BRCA1 haploinsufficiency for replication stress suppression in primary cells
Source: Nat Commun. 2014 Nov 17;5:5496. doi: 10.1038/ncomms6496 (PMC4243249; doi:10.1038/ncomms6496)
Supplement: Supplementary Information — Supplementary Figures 1-7, Supplementary Table 1 and Supplementary References. [file ncomms6496-s1.pdf]

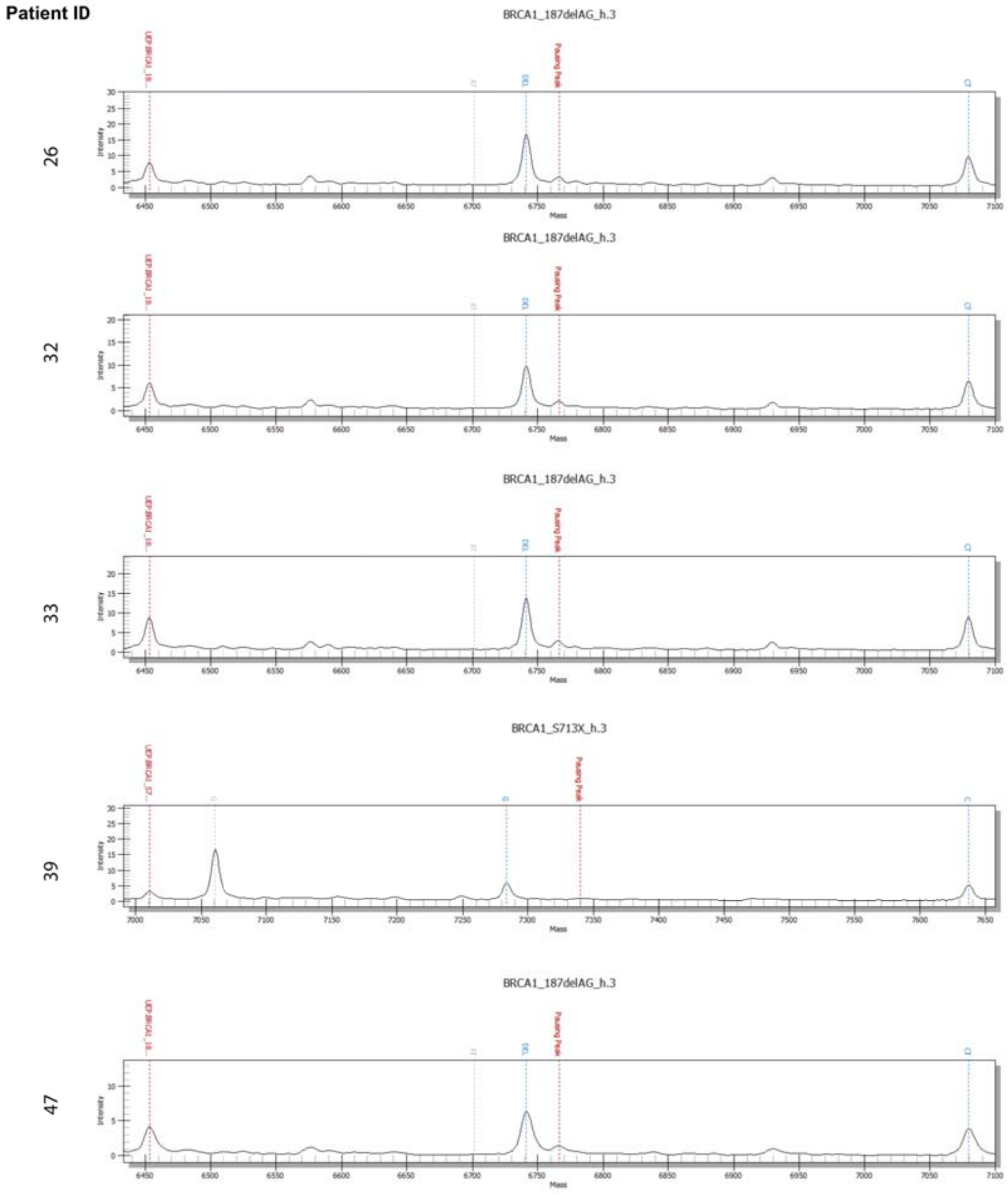

Supplementary Figure 1A, 1B, 1C. Homogeneous Mass- Extend (hME) analysis and DNA sequencing to confirm *BRCA1* mutations in fibroblasts and HMECs. (**1A**) The mutation present in each of the *BRCA1*$^{mut/+}$ $_{fibroblast}$ lines, and one HMEC line (AR1) used in this study were confirmed by homogenous Mass- Extend (hME) analysis. hME profiles of a subset of the mutations is shown, and the rest are available upon request. Genotyping was performed by Sequenom MassARRAY technology (Sequenom Inc., San Diego, CA) using a locus-specific primer extension method, as previously described[1,2]. (**1B, C**) The mutation in each of the *BRCA1*$^{mut/+}$ HMEC strains used in this study was confirmed by direct nucleotide sequencing (Left, L). Corresponding WT sequences are shown on the right.

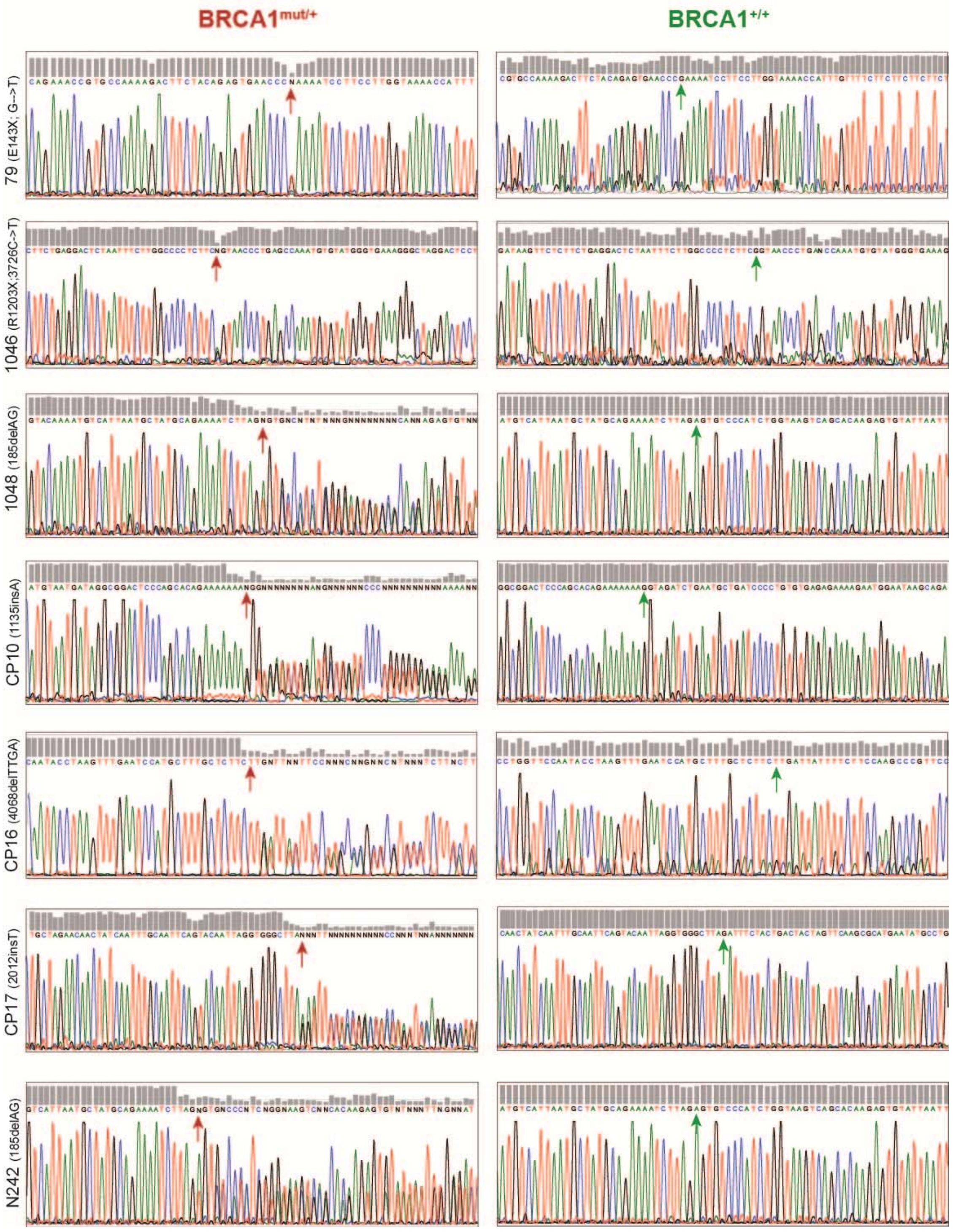

Supplementary Figure 1B

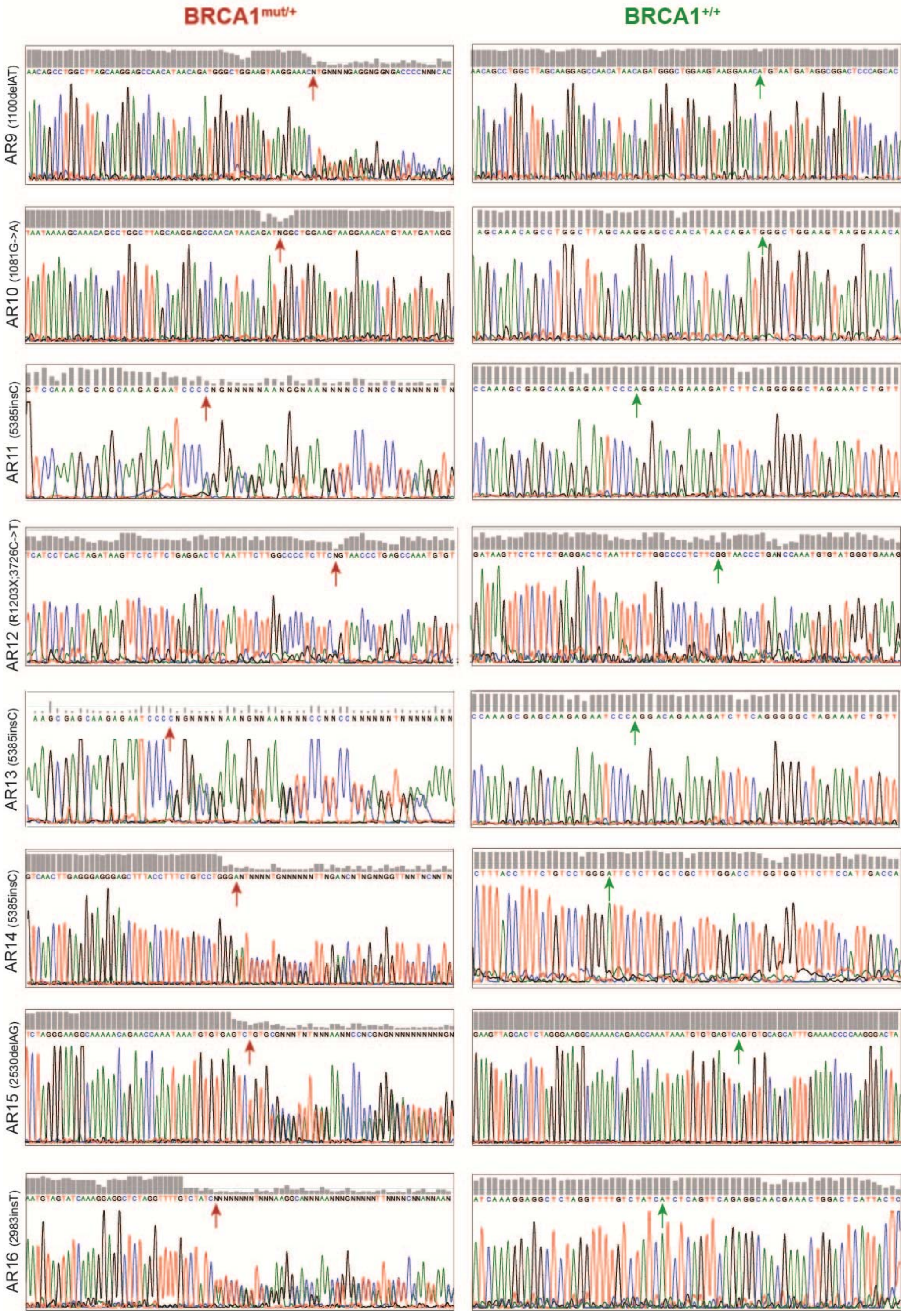

Supplementary Figure 1C

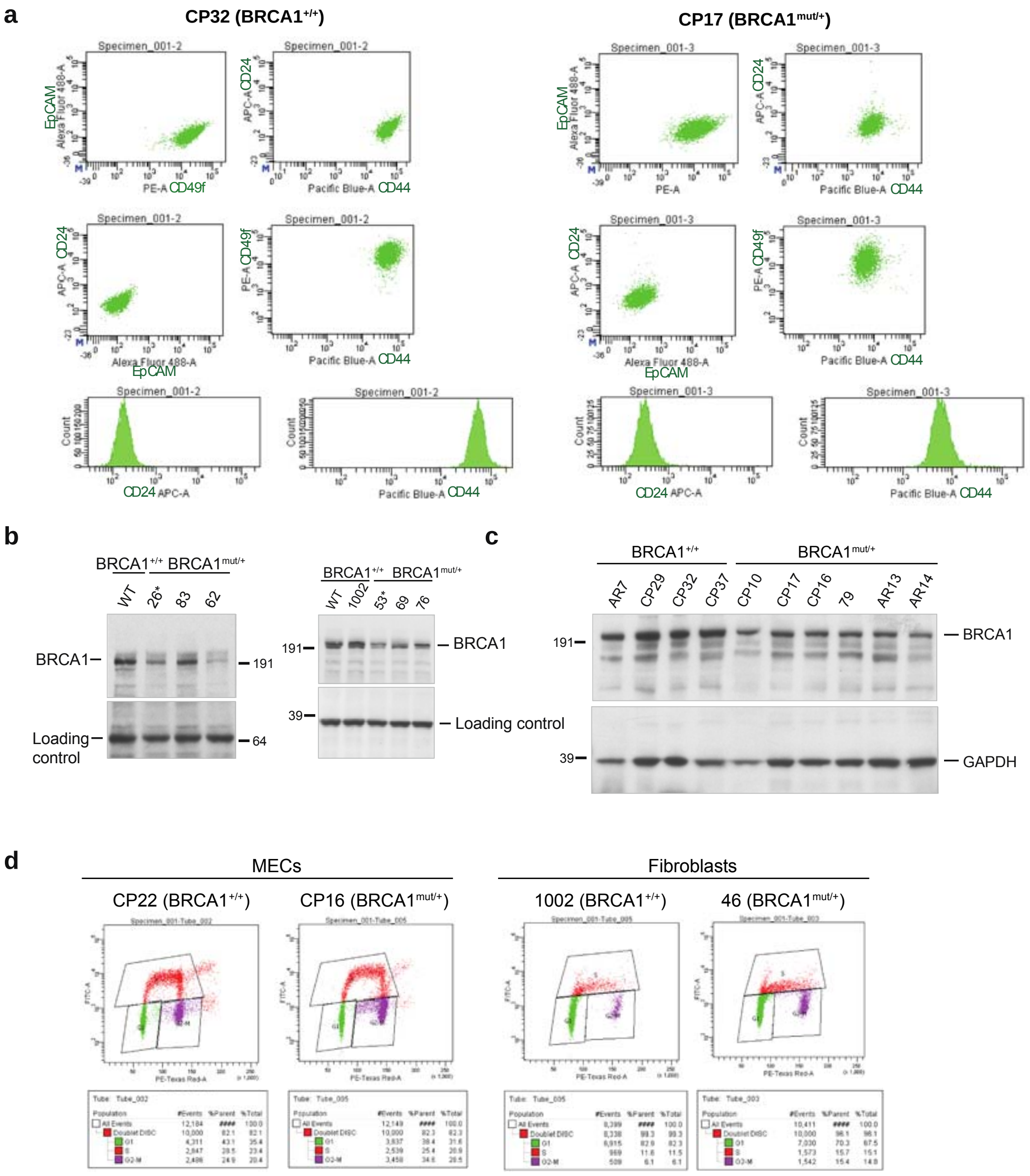

Supplementary Figure 2. BRCA1 protein levels in *BRCA1*$^{mut/+}$ and *BRCA1*$^{+/+}$ cells, and FACS analysis of the cell lineage and replication status of *BRCA1*$^{+/+}$ and *BRCA1*$^{mut/+}$ cells. (**a**) Cell lineage for HMECs was determined by flow cytometry analysis of cell surface marker expression (EpCAM, CD24, CD49f and CD44). This analysis was carried out for the following HMEC strains – *BRCA1*$^{mut/+}$ (CP10, CP16, CP17, AR16, 79 and AR11) and *BRCA1*$^{+/+}$ (CP22, CP29, CP32, AR7). (**b**) Nuclear extracts from *BRCA1*$^{mut/+}$ and *BRCA1*$^{+/+}$ fibroblast strains were prepared and analyzed for BRCA1 protein level. A non-specific band was used as a loading control. (**c**) Whole cell extracts (prepared with NETN300) of HMEC strains were analyzed for BRCA1 protein levels. GAPDH level is used as a loading control. (**d**) Replication profiles for each of the HMEC and fibroblast strains were assayed by BrDU based FACS analysis. Briefly, the cells were pulse- labeled with 10uM BrdU for 30 minutes (for HMECs) and 1.5hours (for fibroblasts-which proliferate more slowly than HMECs) and then fixed for FACS analysis.

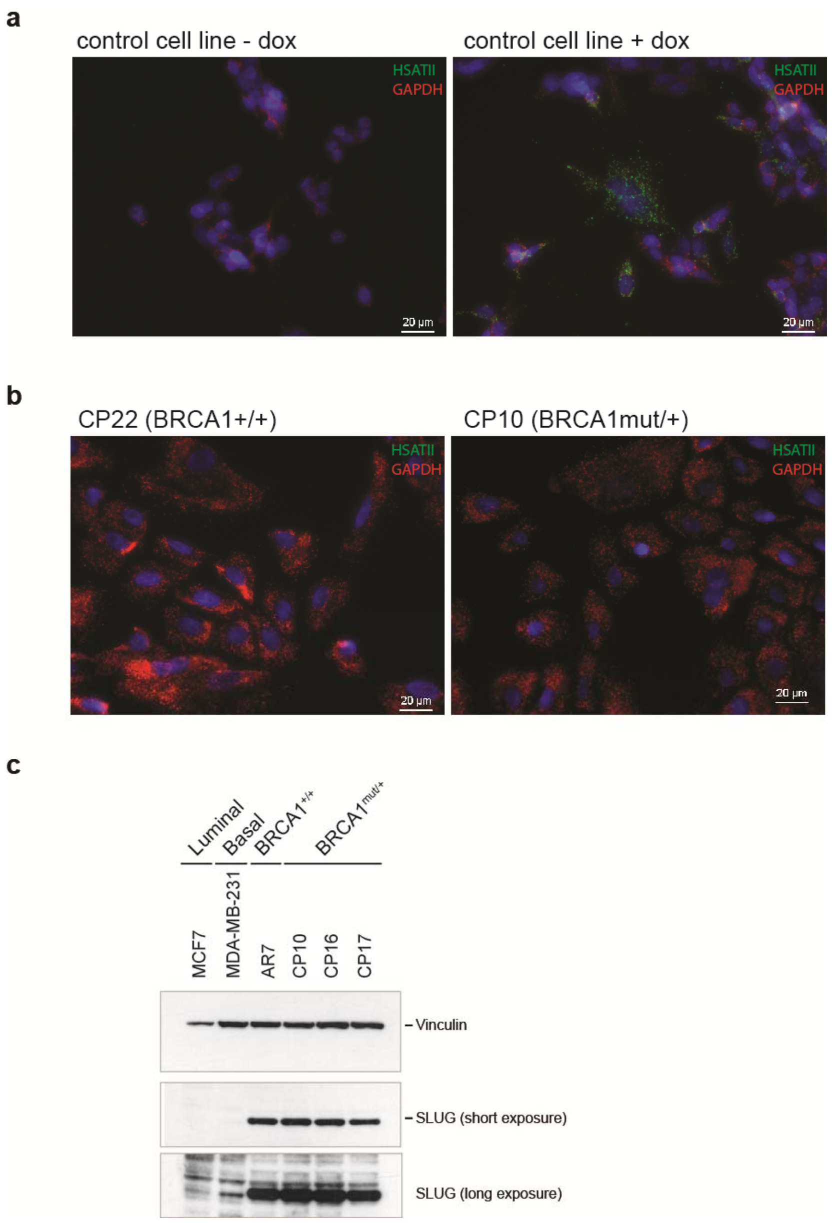

Supplementary Figure 3. Satellite RNA induction and Slug expression in *BRCA1* WT and mutant HMECs. (**a, b**) In situ RNA hybridization was carried out for HSATII in *BRCA1*$^{+/+}$ (CP22, CP32, CP29) and *BRCA1*$^{mut/+}$ (CP10, 79, CP16 and CP17) lines. Images for CP22 and CP10 are shown in the figure. SW620 is a colon cancer line and was used as a positive control for HSATII (it expresses HSATII after dox induction). GAPDH was used as a positive control for RNA FISH in these experiments. (**c**) Steady state levels of Slug in *BRCA1*$^{+/+}$ HMECs (AR7) were similar to those *BRCA1*$^{mut/+}$ strains (CP10, CP16 and CP17). MDA-MB-231 (a basal-like sporadic breast cancer cell line) was used as a positive control here. MCF7 (a luminal line) served as a negative control for SLUG expression. Each panel was taken from the same blot, but the top panel was exposed to vinculin Ab to yield loading control results. The bottom-most panel represents a longer exposure than the middle one. Both reflect SLUG protein abundance.

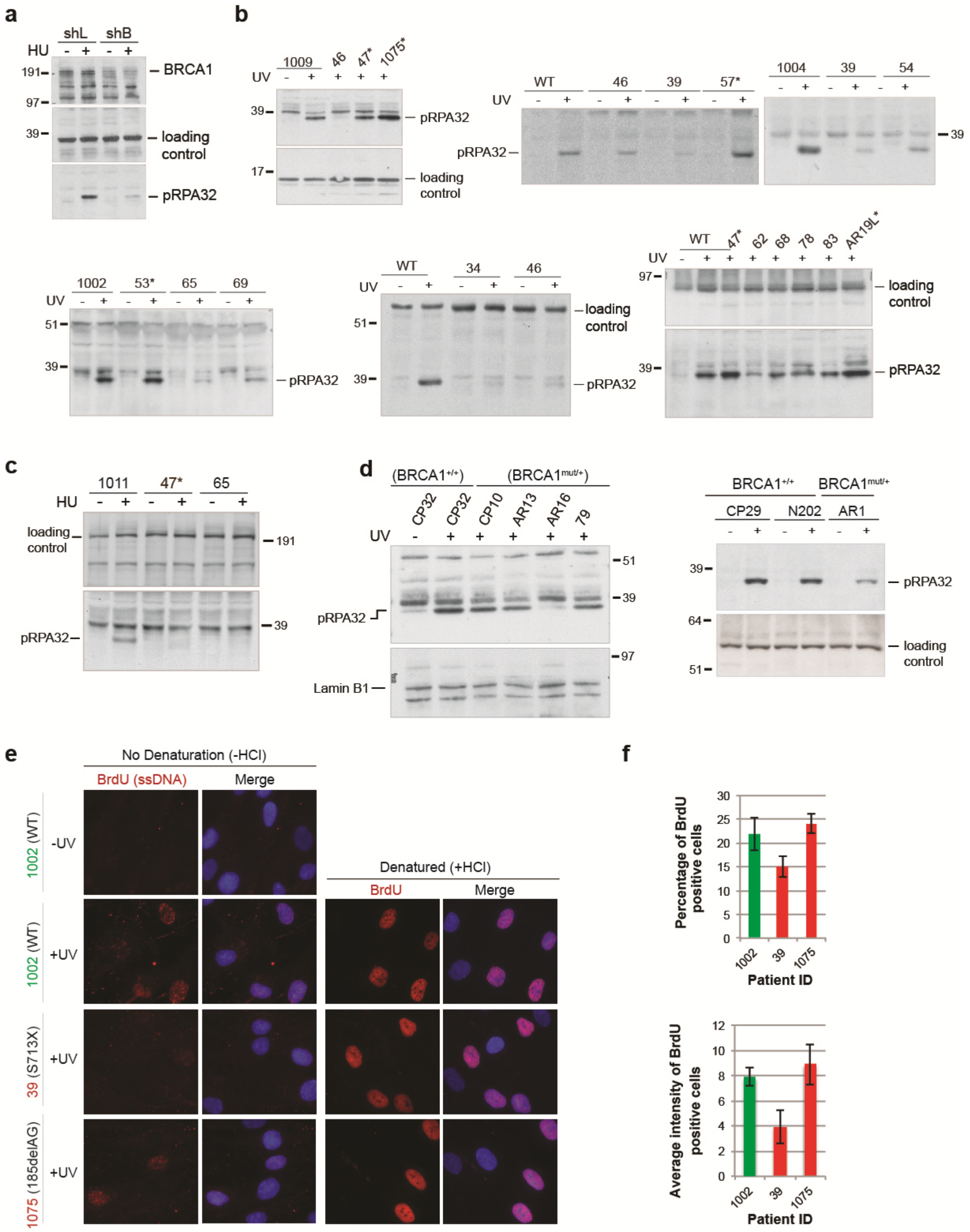
a
shL shB
HU - + - +
191
97
BRCA1
39
loading control
pRPA32
b
1009 46 47* 1075*
UV - + + + +
39
pRPA32
17
loading control
WT 46 39 57*
UV - + - + - + - +
pRPA32
1004 39 54
- + - + - +
39
1002 53* 65 69
UV - + - + - + - +
51
39
pRPA32
WT 34 46
UV - + - + - +
51
loading control
39
pRPA32
WT 47* 62 68 78 83 AR19L*
UV - + + + + + + +
97
loading control
39
pRPA32
c
1011 47* 65
HU - + - + - +
loading control
191
pRPA32
39
d
(BRCA1+/+) (BRCA1mut/+)
CP32 CP32 CP10 AR13 AR16 79
UV - + + + + +
51
39
pRPA32
97
Lamin B1
BRCA1+/+ BRCA1mut/+
CP29 N202 AR1
- + - + - +
39
pRPA32
64
loading control
51
e
No Denaturation (-HCl)
BrdU (ssDNA) Merge
Denatured (+HCl)
BrdU Merge
1002 (WT) -UV
1002 (WT) +UV
39 (S713X) +UV
1075 (185delAG) +UV
f
Percentage of BrdU positive cells
30 25 20 15 10 5 0
1002 39 1075
Patient ID
Average intensity of BrdU positive cells
12 10 8 6 4 2 0
1002 39 1075
Patient ID

Supplementary Figure 4. Generation of ssDNA and pRPA32 loading on chromatin after stalled fork induced DNA damage. (**a**) phospho-RPA32 (pRPA32) loading on chromatin is BRCA1 dependent. U20S cells infected with lentiviral shRNA directed at BRCA1 (ShB) exhibit reduced pRPA32 loading, compared to control infected (ShRNA directed at Luciferase, ShL), after HU -induced stalled fork formation. (**b, c, d**) *BRCA1*$^{mut/+}$ and *BRCA1*$^{+/+}$ fibroblast and HMEC strains were either mock-treated or irradiated with 30J/m^2 of UV and/or exposed to HU (10mM for 3hours). All were harvested 3 hours post damage. Chromatin-rich extracts were prepared and analyzed by western blot for the presence of pRPA32. Each panel represents a different blot. Strains depicted in a given blot replicated similarly on the day of the experiment. Strains marked with asterisk are *185delAG* mutation bearing strains. (**e**) BrdU assay for ssDNA generation after UV- induced stalled replication forks. *BRCA1*$^{+/+}$ (1002) and *BRCA1*$^{mut/+}$ (39 and 1075) fibroblast strains were irradiated with low dose UV (5J/m2) and fixed 4 hrs later to detect the presence of ssDNA. Cells were immunostained for BrdU with or without HCl denaturation of DNA.. Details of the protocol are provided in Materials and Methods. (**f**) Data analyzed in (e) is plotted. Upper panel/chart details percentage of BrdU positive cells in different fibroblast strains. Bottom panel/chart details average intensity of BrdU positive cells as determined by ImageJ software. Error bars represent standard deviation in three independent experiments.

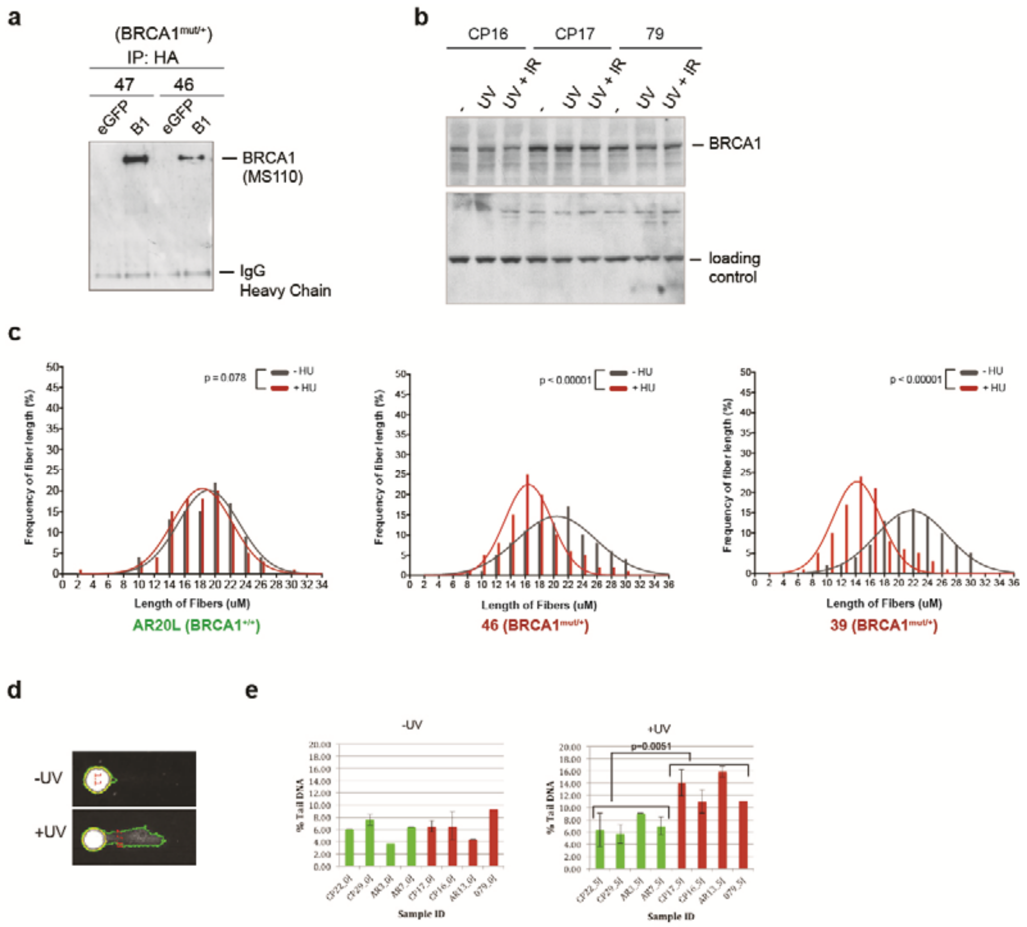

Supplementary Figure 5. The stability of stalled forks is compromised in *BRCA1*$^{mut/+}$ cells. (**a**) *BRCA1*$^{mut/+}$ (47 and 46) were infected with either eGFP expressing or HA-tagged BRCA1 lentiviral vector. Infected cells were grown in presence of Blasticidin. Western blots for the immunoprecipitated (IP) samples were probed with antibody to BRCA1 (MS110). (**b**) CP16, CP17 and 79 (*BRCA1*$^{mut/+}$) cell lines were irradiated with either 15J/m2 UV alone (UV) or with UV followed by 10gy dose of IR (UV + IR). Cells were harvested 4 hours post damage. Protein extracts were analyzed by western blotting for BRCA1. GAPDH served as a loading control in these experiments. (**c**) Distribution of IdU tract lengths, after incubation of cells in presence of HU and/or absence of HU, is plotted as a curve for *BRCA1*$^{+/+}$ fibroblast strains (AR20L) and *BRCA1*$^{mut/+}$ (46 and 39) strains. Experimental design is as described in Fig. 5c. (**d**) *BRCA1*$^{mut/+}$ cells reveal a compromised DNA repair efficiency compared to *BRCA1*$^{+/+}$ cells after exposure to low dose UV (5J/m2). DNA damage was measured as a percentage of DNA in comet tails after UV. Representative images for comets in unirradiated (-UV) and irradiated (+UV) samples are shown. (**e**) *BRCA1*$^{mut/+}$ and *BRCA1*$^{+/+}$ HMECs were irradiated with 5J/m2 of UV and allowed to recover for 3 hours before carrying out the alkaline comet assay. Percentage of DNA in the comet tails is plotted for unirradiated (left panel) and irradiated cells (right panel). Green bars = *BRCA1*$^{+/+}$ and red= *BRCA1*$^{mut/+}$ cells. The mean result and standard deviation of at least three different experiments is plotted. In each experiment at least 250 individual cells were scored for percentage of DNA in the comet tails using CellProfiler software.

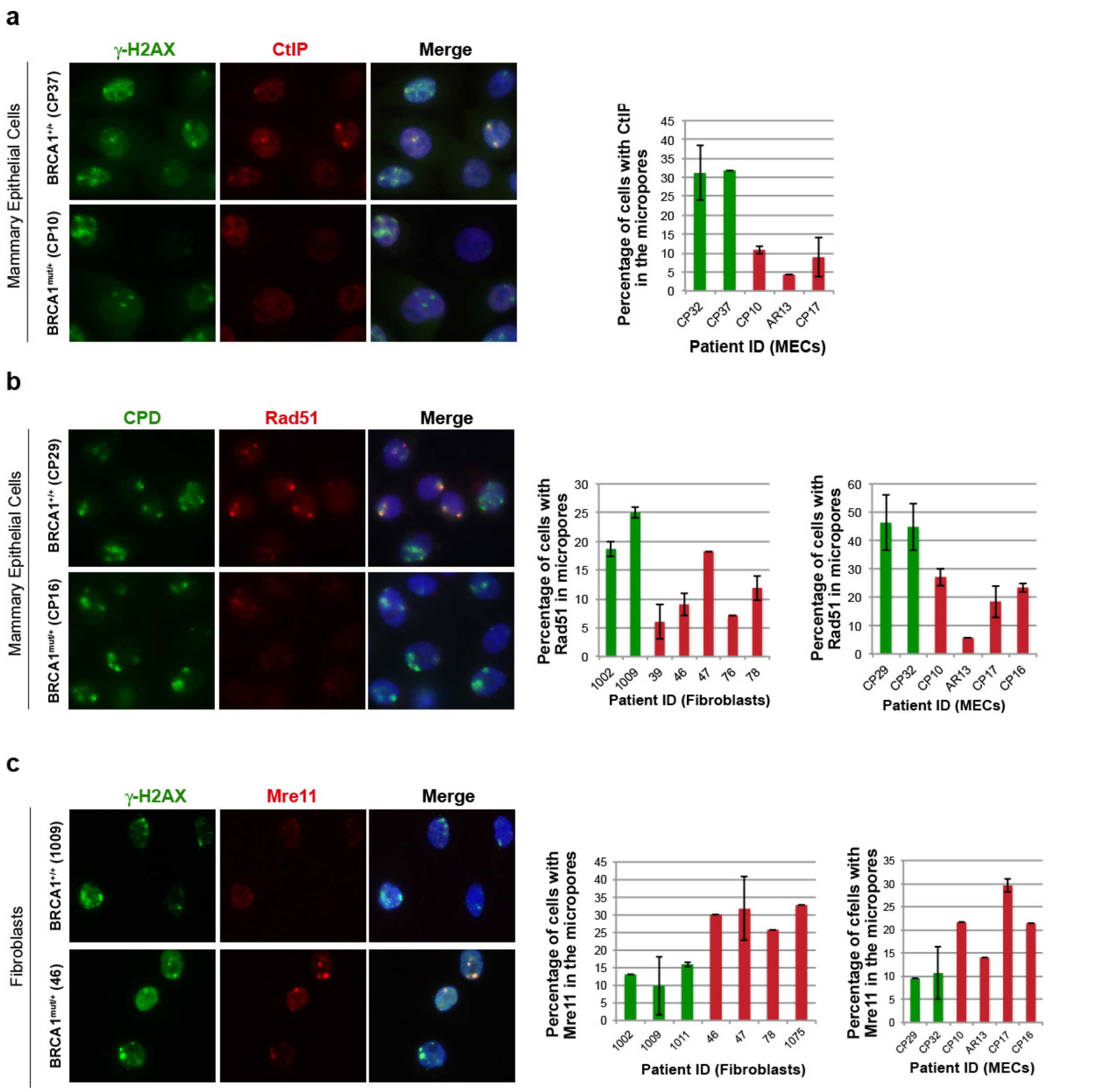

Supplementary Figure 6. Recruitment of CtIP, Rad51 and Mre11 to sites of stalled forks (after UV- induced DNA damage) in *BRCA1*$^{+/+}$ and *BRCA1*$^{mut/+}$ strains. *BRCA1*$^{+/+}$ and *BRCA1*$^{mut/+}$ HMECs and fibroblast strains were irradiated with 30J/m2 UV through micropore filters. Cells were fixed 3 hours post UV- induced DNA damage and immunostained for CtIP (**a**), Rad51 (**b**) and Mre11 (**c**). CPD (cyclobutane pyrimidine dimers) and γ-H2AX staining was used to mark the sites of UV damage/stalled forks. Plots on the right show percentage of cells with the respective proteins (CtIP, Rad51 and/or Mre11) localized in micropores. Green bars denote *BRCA1*$^{+/+}$ strains and red bars denote *BRCA1*$^{mut/+}$ strains in all the plots.

Ful Scans of western blots in Figure 1

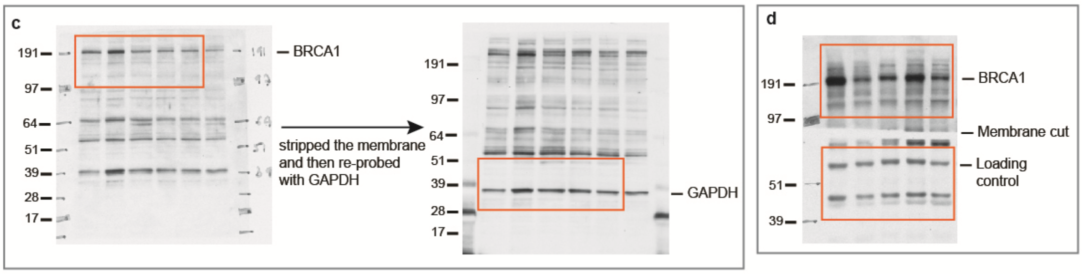

Ful Scans of western blots in Figure 4

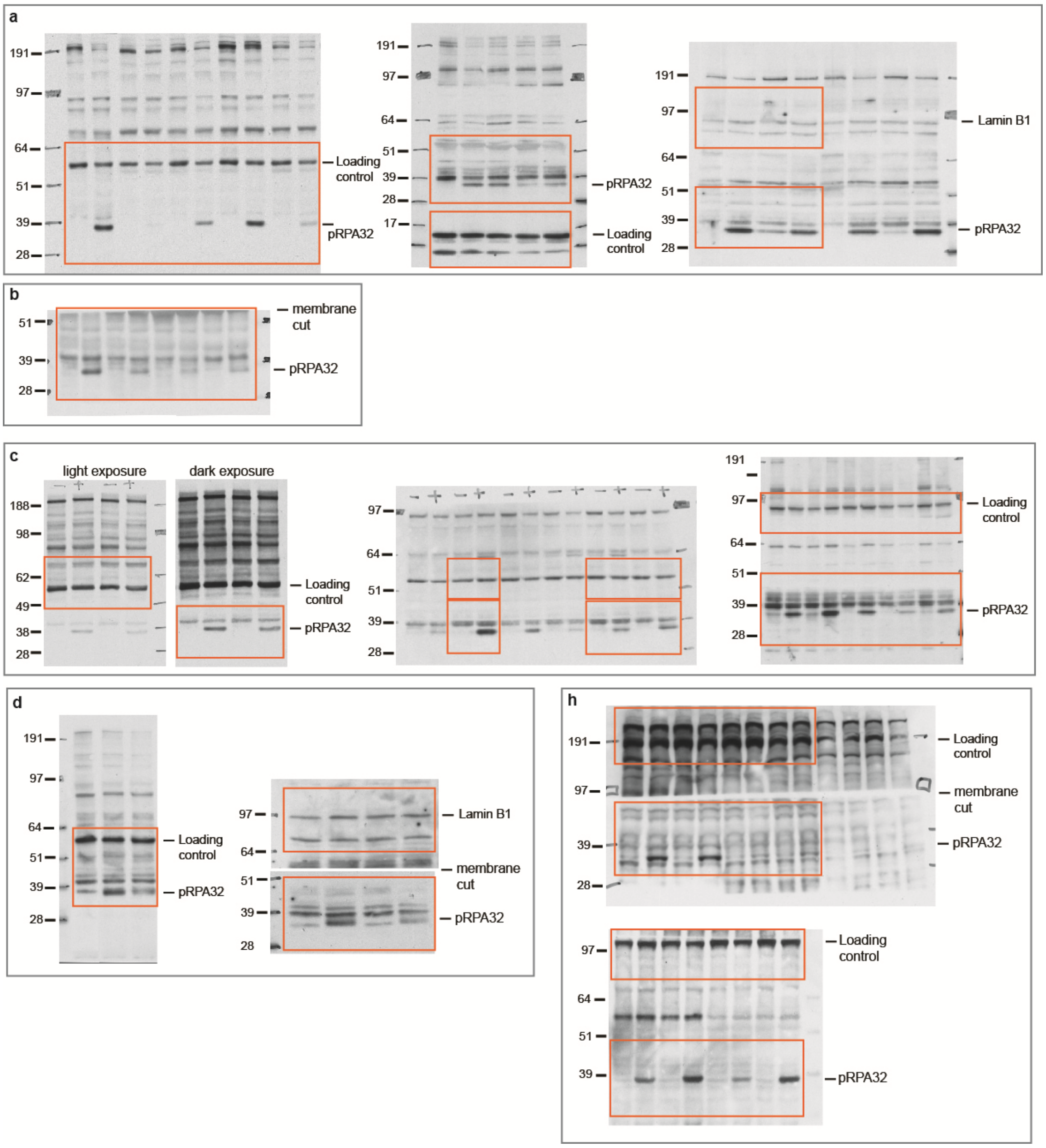

Supplementary Figure 7. Full scans of western blots in the main text and in supplementary section

Ful Scans of western blots in Figure 4 (contd.)

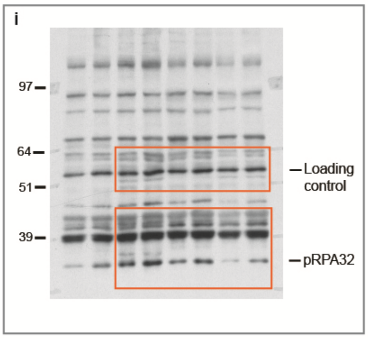

Ful Scans of western blots in Figure 6

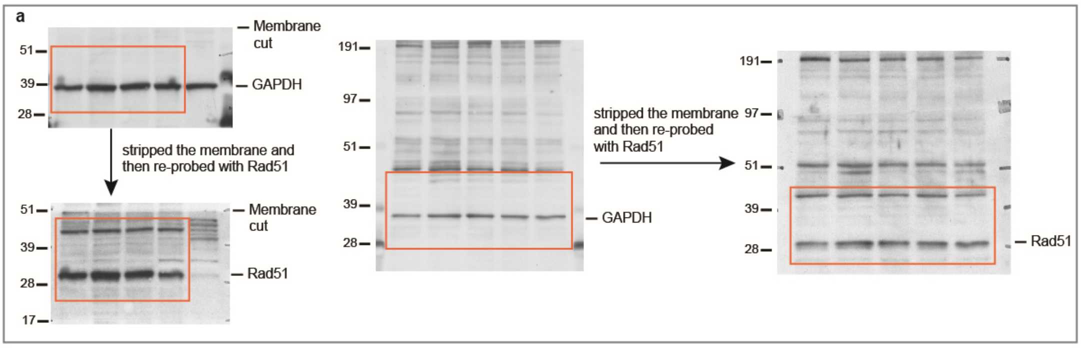

Ful Scans of western blots in Supplemental Figure 2

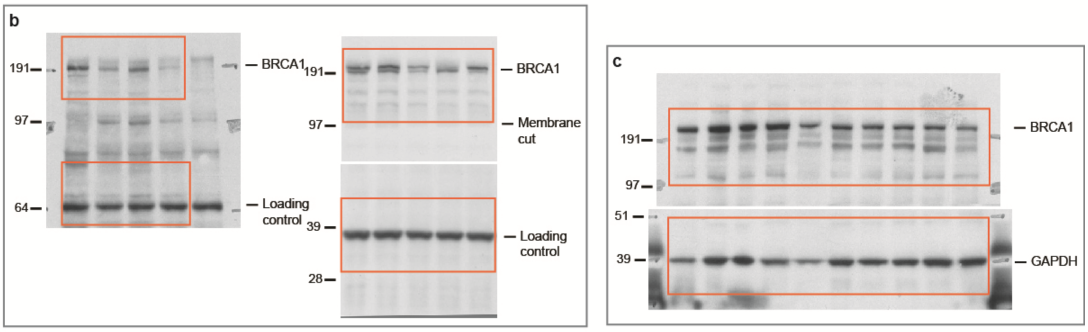

Ful Scans of western blots in Supplemental Figure 3

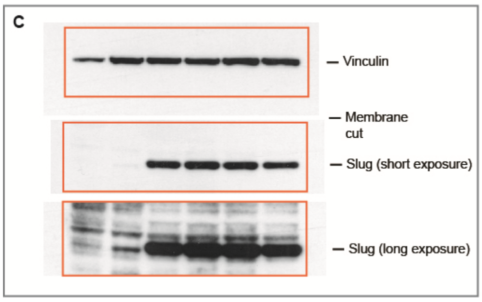

Supplementary Figure 7. Full scans of western blots in the main text and in supplementary section

Ful Scans of western blots in Supplemental Figure 4.

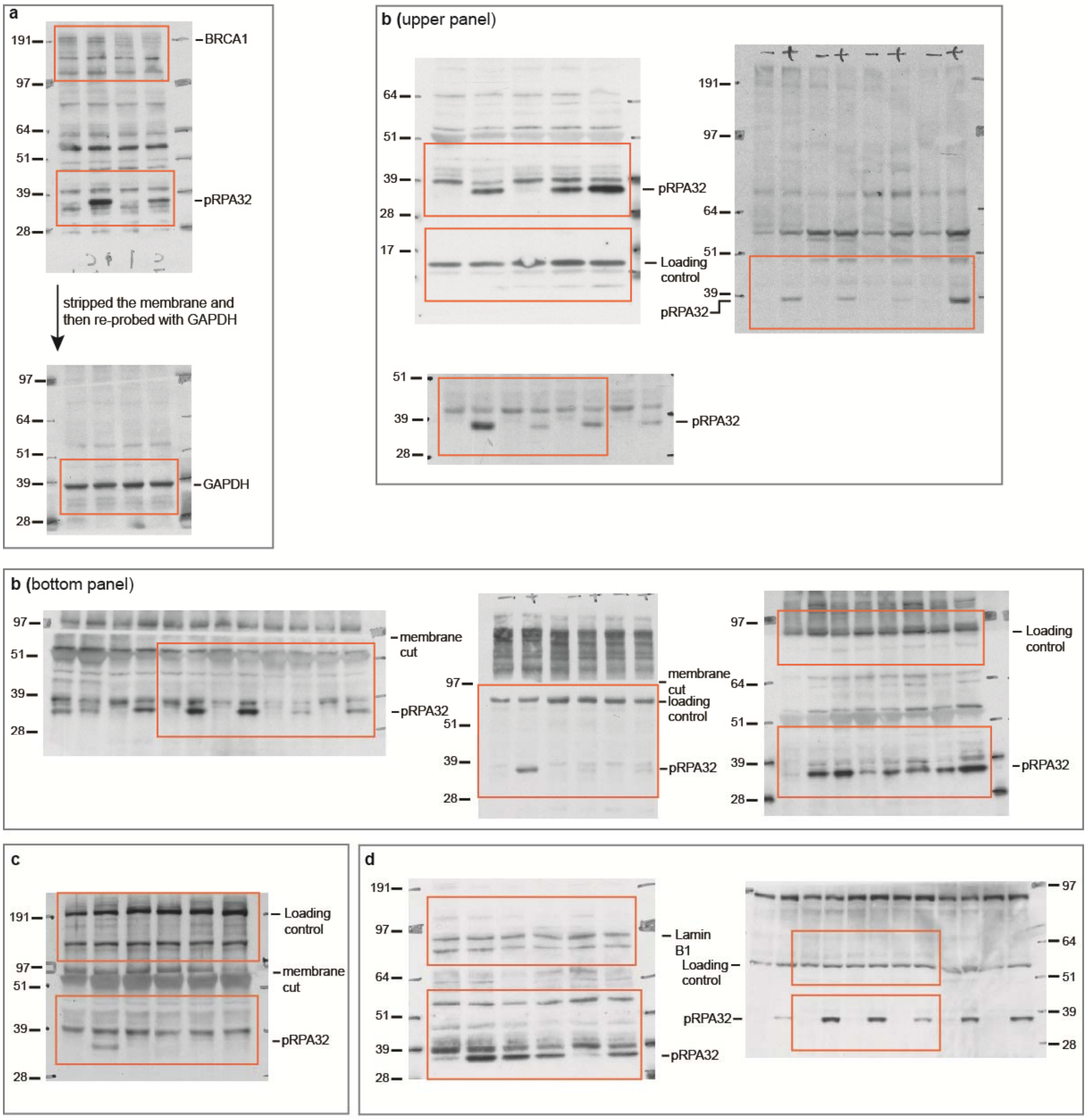

Ful Scans of western blots in Supplemental Figure 5

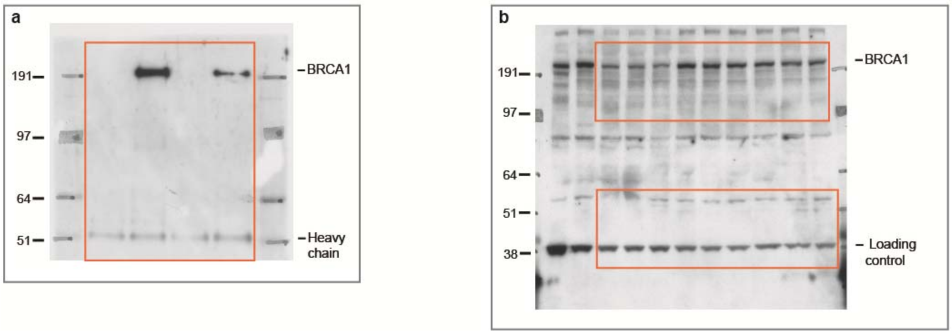

Supplementary Figure 7. Full scans of western blots in the main text and in supplementary section

Supplementary Table 1. Summary of results. Results of different assays performed with all the WT and *BRCA1* mutation bearing fibroblasts and HMECs are summarized in table 1. ND = Not Determined.

Cell Type	Number	Study ID	BRCA1 Mutation	RPA loading after UV/HU	Collapsed Forks	Rad51 at DSBs	Spindle Formation	Centrosome Number
Fibroblast	1	26	185delAG	Yes	Yes	Yes	Yes	≤2
Fibroblast	2	32	185delAG	Yes	Yes	Yes	Yes	≤2
Fibroblast	3	33	185delAG	Yes	Yes	Yes	Yes	≤2
Fibroblast	4	34	Y1463X	Defective	Yes	Yes	Yes	≤2
Fibroblast	5	39	S713X	Defective	Yes	Yes	Yes	≤2
Fibroblast	6	45	5083del19	Defective	Yes	Yes	Yes	≤2
Fibroblast	7	46	1137delG	Defective	Yes	Yes	Yes	≤2
Fibroblast	8	47	185delAG	Yes	Yes	Yes	Yes	≤2
Fibroblast	9	48	4184del4	Defective	Yes	Yes	Yes	≤3
Fibroblast	10	53	185delAG	Yes	ND	ND	ND	ND
Fibroblast	11	54	4154delA	Defective	Yes	ND	ND	ND
Fibroblast	12	57	185delAG	Yes	Yes	Yes	Yes	≤2
Fibroblast	13	62	1294del40	Defective	Yes	Yes	Yes	≤2
Fibroblast	14	65	3819del5	Defective	Yes	ND	ND	ND
Fibroblast	15	68	Q491X (1590C>T)	Defective	Yes	Yes	Yes	≤2
Fibroblast	16	69	5385insC	Defective	Yes	Yes	Yes	≤2
Fibroblast	17	73	795delT	ND	Yes	ND	ND	ND
Fibroblast	18	76	2530delAG	Defective	Yes	Yes	Yes	≤2
Fibroblast	19	78	W1815X	Defective	Yes	Yes	Yes	≤2
Fibroblast	20	80	185insA	Defective	Yes	Yes	Yes	≤2
Fibroblast	21	82	185delAG	Defective	Yes	ND	Yes	≤2
Fibroblast	22	83	IVS19+1G>A	Defective	Yes	Yes	Yes	≤2
Fibroblast	1	WT	N/A	Yes	No	Yes	Yes	≤2
Fibroblast	2	1002	N/A	Yes	No	Yes	Yes	≤2
Fibroblast	3	1004	N/A	Yes	No	ND	Yes	≤2
Fibroblast	4	1006	N/A	Yes	No	Yes	Yes	≤2
Fibroblast	5	1007	N/A	ND	No	Yes	Yes	≤2
Fibroblast	6	1008	N/A	ND	No	Yes	Yes	≤2
Fibroblast	7	1009	N/A	ND	No	Yes	Yes	≤2
Fibroblast	8	1010	N/A	Yes	No	Yes	Yes	≤2
Fibroblast	9	1011	N/A	Yes	No	Yes	Yes	≤2
Fibroblast	10	AR8F	N/A	Yes	No	Yes	Yes	≤2

MEC	1	79	E143X	Defective	Yes	Yes	Yes	≤2
MEC	2	1046	3725C>T (R1203X)	ND	Yes	ND	ND	ND
MEC	3	1048	187delAG	ND	Yes	Yes	Yes	≤2
MEC	4	CP10	1135insA	Defective	Yes	Yes	Yes	≤2
MEC	5	CP16	4065-4068del	Defective	Yes	Yes	Yes	≤2
MEC	6	CP17	2012insT	Defective	Yes	Yes	Yes	≤2
MEC	7	AR1	R1443X	ND	ND	ND	ND	ND
MEC	8	AR9	1100delAT	ND	Yes	ND	ND	ND
MEC	9	AR10	1081G->A	ND	ND	Yes	Yes	Yes
MEC	10	AR11	5385insC	ND	Yes	ND	ND	ND
MEC	11	AR12	R1203X	ND	Yes	ND	ND	ND
MEC	12	AR13	5385insC	ND	Yes	Yes	Yes	Yes
MEC	13	AR14	5385insC	ND	Yes	Yes	Yes	Yes
MEC	14	AR15	2530delAG	ND	Yes	ND	ND	ND
MEC	15	AR16	2983insT	ND	Yes	Yes	Yes	Yes
MEC	1	CP14	N/A	ND	No	Yes	Yes	≤2
MEC	2	CP22	N/A	Yes	No	Yes	Yes	≤2
MEC	3	CP29	N/A	Yes	No	Yes	Yes	≤2
MEC	4	CP32	N/A	Yes	No	Yes	Yes	≤2
MEC	5	AR4	N/A	ND	No	Yes	Yes	≤2
MEC	6	AR7	N/A	ND	No	Yes	Yes	≤2
MEC	7	N202	N/A	Yes	No	Yes	ND	ND

Supplementary References:

1. MacConaill, L., *et al.* Profiling critical cancer gene mutations in clinical tumor samples. *PLOS ONE* **4**, e7887 (2009).
2. Thomas, R.K., *et al.* High-throughput oncogene mutation profiling in human cancer. *Nature genetics* **39**, 347-351 (2007).
